# Synthetic Routes to *N*-9 Alkylated 8-Oxoguanines; Weak Inhibitors of the Human DNA Glycosylase OGG1

**DOI:** 10.3390/molecules200915944

**Published:** 2015-09-02

**Authors:** Tushar R. Mahajan, Mari Eknes Ytre-Arne, Pernille Strøm-Andersen, Bjørn Dalhus, Lise-Lotte Gundersen

**Affiliations:** 1 Department of Chemistry, University of Oslo, P. O. Box 1033, Blindern, N-0315 Oslo, Norway; E-Mail: t.r.mahajan@kjemi.uio.no; 2 Department of Microbiology, Oslo University Hospital, P. O. Box 4950, Nydalen, N-0424 Oslo, Norway; E-Mails: Mari.Ytre-Arne@rr-research.no (M.E.Y.-A.); Bjorn.Dalhus@medisin.uio.no (B.D.); 3 Department of Medical Biochemistry, Institute of Clinical Medicine, University of Oslo, P. O. Box 4950, Nydalen, N-0424 Oslo, Norway; E-Mail: pernille.strom-andersen@medisin.uio.no

**Keywords:** alkylation, cancer, DNA, enzyme inhibitors, guanine, halogenation

## Abstract

The human 8-oxoguanine DNA glycosylase OGG1 is involved in base excision repair (BER), one of several DNA repair mechanisms that may counteract the effects of chemo- and radiation therapy for the treatment of cancer. We envisage that potent inhibitors of OGG1 may be found among the 9-alkyl-8-oxoguanines. Thus we explored synthetic routes to 8-oxoguanines and examined these as OGG1 inhibitors. The best reaction sequence started from 6-chloroguanine and involved *N*-9 alkylation, *C*-8 bromination, and finally simultaneous hydrolysis of both halides. Bromination before *N*-alkylation should only be considered when the *N*-substituent is not compatible with bromination conditions. The 8-oxoguanines were found to be weak inhibitors of OGG1. 6-Chloro-8-oxopurines, byproducts in the hydrolysis of 2,6-halopurines, turned out to be slightly better inhibitors than the corresponding 8-oxoguanines.

## 1. Introduction

Chemo- and radiotherapy are, in addition to surgery for removal of solid tumors, the two main treatment protocols currently available to improve the outcome of cancer patients in general, but treatment-related toxicity, the risk of secondary cancers, and the emergence of resistance limit their effectiveness [1]. Some chemotherapeutic drugs and radiotherapy work partly by imposing high concentrations of DNA damage on the genome of cancer cells, beyond the repair capacity of those cells. The drug-exposed cancer cells are heavily dependent on efficient DNA repair to survive. Consequently, inhibitors that reduce DNA repair activities should sensitize cancer cells to chemo- and/or radiotherapy [2,3,4,5].

**Figure 1:**
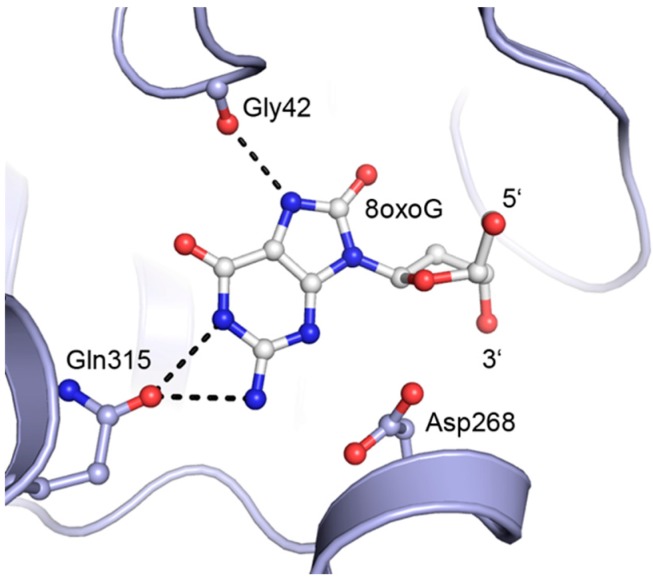
Structural details of 8oxoG base flipped into the lesion recognition pocket of OGG1 (Protein Data Bank deposition 1EBM [7]). The protein backbone is shown as a blue ribbon/helix. Selected amino acid side chains and the 8oxoG base are shown as ball-and-stick. Hydrogen bonds between the protein and 8oxoG are shown as dashed lines. Asp268 is the catalytic residue in OGG1. Symbols 5′ and 3′ indicate the position of the 5′ and 3′ phosphodiester links in the DNA.

Several DNA repair mechanisms counteract exogenous and endogenous processes that destabilize or directly damage genomes. The processes include, among others, base excision repair (BER), a mechanism that depends on enzymes that recognize small modifications in the native bases in DNA, resulting from alkylation, oxidation, deamination, or hydrolysis of the DNA bases. The pathway is initiated by a damage-specific DNA glycosylase that removes the altered base [6]. Some of these enzymes mainly remove oxidized bases, such as the human *8*-*oxoguanine DNA glycosylase* (OGG1) that removes guanines that have been oxidized at the C8-position. The 8-oxoguanine base in the DNA is flipped into a lesion recognition pocket on the enzyme surface, exposing the Watson–Crick signature of guanine and the oxidized C8 position (Figure 1).

We envisage that potent inhibitors of OGG1 may be found among the 9-alkyl-8-oxoguanines. The 8-oxo derivatives of guanosine or deoxyguanosine are probably not inhibitors of the glycosylases since they themselves may be substrates for the enzymes that cleave *N*,*O*-acetals in nucleic acids. As a continuance of our synthetic studies directed towards 9-substituted 8-oxoadenines [8,9], we herein present strategies for the synthesis of *N*-9 substituted 8-oxoguanines. Previous routes include rather tedious constructions of the guanine ring system [10,11,12], and hydrolysis of purine precursors; hydrolysis of 8-halopurines [13,14,15,16], or less conveniently hydrolysis of *N*-7 functionalizedpurines [11,17,18,19]. Results regarding inhibitory activity against the human DNA glycosylase OGG1 are also presented.

## 2. Results and Discussion

### 2.1. Chemistry

**Scheme 1:**
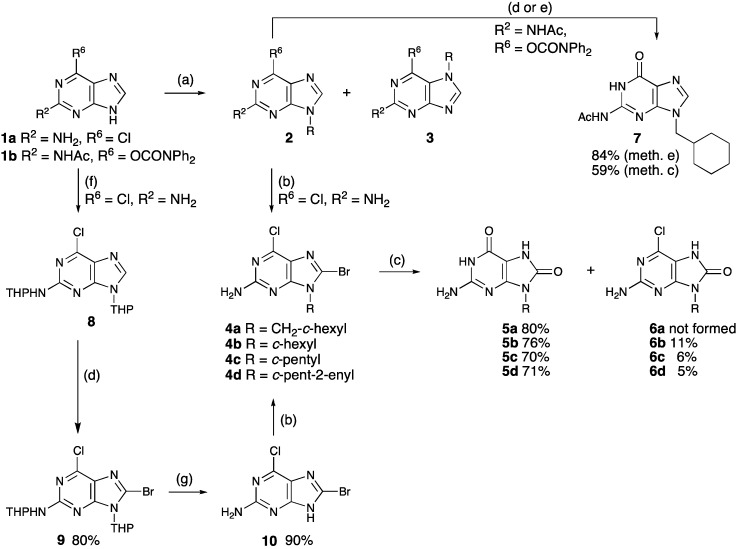
Synthetic routes to 8-oxoguanines **5**.

We found it most convenient to start the synthesis of 9-alkyl-8-oxopurines from commercially available purines, and in our opinion the best way to introduce the 8-oxo group would be by hydrolysis of an 8-halopurine. However, there still was the question of whether the halogen or the *N*-9 substituent should be introduced first and which protection/activation groups should be employed in the synthesis. Ideally, such groups should also be removed in the final hydrolysis step. Regioselectivity in *N*-alkylation of guanine derivatives was also an issue [20,21,22,23,24,25,26,27]. We chose to start from two guanine precursors, commercially available 2-amino-6-chloropurine (**1a**) and the *O*-carbamoylguanine **1b**, easily available from guanine [28,29]. The synthetic routes explored are all summarized in Scheme 1.

*Reagents and conditions*: (**a**) See Table 1; (**b**) See Table 2; (**c**) 1. Ac_2_O, NaOAc, AcOH, 2. NaOH(aq), ∆; (**d**) 1. LDA, 2. (CCl_2_Br)_2_, THF, −78 °C; (**e**) Br_2_, CHCl_3_; (**f**) See [30]; (**g**) HCl(aq), EtOH.

**Table 1:** *N*-alkylation of guanine precursors **1a** and **1b**.

Entry	R^2^	R^6^	R	Reagents and Conditions	Ratio 2:3:1 ^a^	Yield (%) 2 ^b^	Yield (%) 3 ^b^
1	Cl	NH_2_	CH_2_-*c*-hexyl	RBr, K_2_CO_3_, DMF, rt, 72 h	80:20:0	67, **2a**	10, **3a**
2	Cl	NH_2_	CH_2_-*c*-hexyl	ROH, DIAD, PPh_3_, THF, 70 °C, 14 h	93:7:0	76, **2a**	5, **3a**
3	OCONPh_2_	NHAc	CH_2_-*c*-hexyl	RBr, K_2_CO_3_, DMF, rt, 72 h	81:19:0	45, **2e**	7, **3e**
4	OCONPh_2_	NHAc	CH_2_-*c*-hexyl	ROH, DIAD, PPh_3_, THF, 70 °C, 14 h	82:18:0	70, **2e**	3, **3e**
5	Cl	NH_2_	*c*-hexyl	RI, K_2_CO_3_, DMF, rt, 72 h	15:0:85	– ^c^	–
6	Cl	NH_2_	*c*-hexyl	ROTs, K_2_CO_3_, DMF, rt, 72 h	– ^d^	33, **2b**	– ^c^
7	Cl	NH_2_	*c*-hexyl	ROH, DIAD, PPh_3_, THF, 70 °C, 14 h	8:4:88	– ^c^	– ^c^
8	Cl	NH_2_	*c*-hexyl	ROH, DIAD, PPh_3_, THF, ultrasound, 14 h	27:0:73	20, **2b**	–
9	Cl	NH_2_	*c*-hexyl	ROH, DIAD, PPh_3_, DMF, 150 °C, μW, 2 h	41:8:51	– ^c^	– ^c^
10	OCONPh_2_	NHAc	*c*-hexyl	ROTs, K_2_CO_3_, THF, rt, 72 h	– ^d^	30, **2f**	– ^c^
11	OCONPh_2_	NHAc	*c*-hexyl	ROTs, K_2_CO_3_, DMF, 80 °C, 72 h	– ^d,e^	– ^c^	– ^c^
12	OCONPh_2_	NHAc	*c*-hexyl	ROH, DIAD, PPh_3_, THF, 70 °C, 14 h	– ^d^	22, **2f**	– ^c^
13	Cl	NH_2_	*c*-pentyl	RBr, K_2_CO_3_, DMF, rt, 72 h	86:14:0	71, **2c**	5, **3c**
14	Cl	NH_2_	*c*-pentyl	ROH, DIAD, PPh_3_, THF, 70 °C, 14 h	91:9:0	72, **2c**	6, **3c**
15	OCONPh_2_	NHAc	*c*-pentyl	RBr, K_2_CO_3_, DMF, rt, 72 h	76:15:09	52, **2g**	– ^c^
16	OCONPh_2_	NHAc	*c*-pentyl	ROH, DIAD, PPh_3_, THF, 70 °C, 14 h	90:10:0	58, **2g**	– ^c^
17	Cl	NH_2_	*c*-pent-2-enyl	RBr, K_2_CO_3_, DMF, rt, 24 h	23:16:61	18, **2d**	– ^c^
18	Cl	NH_2_	*c*-pent-2-enyl	ROH, DIAD, PPh_3_, THF, 70 °C, 42 h	55:18:27	40, **2d**	– ^c^
19	Cl	NH_2_	*c*-pent-2-enyl	ROAc, Pd(PPh_3_)_4_,NaH, DMSO, ^f^ 50 °C, 48 h	75:25:0	53, **2d**	18, **3d**

^a^ From ^1^H-NMR spectra of the crude products, the signals from H-8 in compounds **1**, **2**, and **3** were integrated; ^b^ Isolated yields; ^c^ Not isolated in pure form; ^d^ Difficult to determine due to overlapping signals in the ^1^H-NMR spectra; ^e^ A complex mixture was formed; ^f^ Comparable results were obtained in DMF.

**Table 2:** Synthesis of 8-bromopurines **4**.

Entry	Starting Material ^a^	Reagents and Conditions	Yield (%) 4 ^a,b^
1	**2a**	Br_2_, H_2_O	79%, **4a**
2	**10**	RBr, K_2_CO_3_, DMF	34%, **4a**
3	**10**	ROH, DIAD, PPh_3_, THF, 70 °C	56%, **4a**
4	**2b**	Br_2_, H_2_O	70%, **4b**
5	**2c**	Br_2_, H_2_O	81%, **4c**
6	**2d**	1. LDA, 2. CCl_2_BrCCl_2_Br, THF, −78 °C	32%, **4d**
7	**10**	ROH, DEAD, PPh_3_, THF, 70 °C	42%, **4d**
8	**10**	ROAc, Pd(PPh_3_)_4_, NaH, DMF, 50 °C	29%, **4d**

^a^ The structures are shown in Scheme 1; ^b^ Isolated yields.

First we chose to *N*-alkylate the substrates **1** before *C*-8 halogenation and hydrolysis. Alkylations were conducted by various methodologies in order to find the conditions that gave the desired *N*-9 alkylated isomer **2** with high selectivity and in a good isolated yield (Scheme 1, Table 1). Relatively simple alkylating agents were chosen for the model reactions and we focused on alkylation with alkyl halides in the presence of base, Mitsunobu reactions, and Pd-catalyzed allylic alkylation.

The cyclohexylmethyl substituent could be introduced at *N*-9 either by reaction with alkyl bromide in the presence of a base [31,32] (Table 1, Entries 1 and 3) or with cyclohexylmethanol under Mitsunobu conditions (Table 1, Entries 2 and 4). The latter is often claimed to be more *N*-9 selective compared to classical alkylations of purines [33,34,35]. In all cases a mixture of the *N*-9 and *N*-7 alkylated isomers (**2** and **3**) was formed with good selectivity for the desired isomer **2**. The isomers were identified from HMQC and HMBC-NMR, as described before [31].

The guanine precursor **1b**, carrying a bulky substituent at *C*-6 that may sterically block *N*-7, is reported to react with high *N*-9 selectivity in other *N*-functionalization reactions [28,29,36,37,38,39,40,41]. Nevertheless, we found the regioselectivity in *N*-alkylation of purine **1b** equal or slightly poorer compared to 6-chloroguanine **1a** in all reactions performed in this study. In the alkylation of compound **1b**, minor amounts of other relatively polar products were formed under both reaction conditions. These often made purification of the *N*-7 alkylated isomer **3** difficult. The identity of the byproducts could not be determined, but they may be formed as a result of cleavage of the O^6^-protecting group. Alkylation of N^2^, as observed by others [41], was not seen.

Introduction of the cyclohexyl group at *N*-9 turned out to be quite difficult (Table 1, Entries 5–12). Both starting materials (**1a** and **1b**) did not react with cyclohexyl bromide (data not shown) and reacted slowly with cyclohexyl iodide or the corresponding tosylate, but compounds **2b** and **2f** could be isolated in modest yields (Table 1, Entries 5, 6, 10 and 11). It is, however, well known that cyclohexyl halides or pseudo halides may react sluggishly in substitution reactions [42]. The results were not significantly improved when the Mitsunobu reaction was employed (Table 1; Entries 7, 8, and 12), not even under ultrasound (Table 1, Entry 8) or microwave conditions (Table 1, Entry 9).

The cyclopentyl group could easily be installed at *N*-9 on both starting materials **1a** and **1b** by reaction with cyclopentyl bromide and base (Table 1, Entries 13 and 15) or by alkylation under Mitsunobu conditions (Table 1, Entries 14 and 16). The selectivity for *N*-9 was higher in the Mitsunobu reactions, but the isolated yields were comparable due to more tedious purification when Mitsunobu conditions, also producing phosphine oxides and reduced azodicarboxylates, were employed.

Finally we introduced the cyclopent-2-enyl group at *N*-9 (Table 1, Entries 17–19). These reactions were only conducted at the guanine precursor **1a**, since we so far had not observed any significant improvement in regioselectivity when compound **1b** was employed and we had observed problems with compounds derived from purine **1b** later in the planned synthetic sequence. In addition to alkylation with the halide and Mitsunobu reaction with the alcohol, we also attempted palladium catalyzed alkylation with the allylic acetate [43]. 3-Bromocyclopentene could only be generated as a 15% solution in CCl_4_ and the reagent had a limited stability, probably partly due to traces of the radical initiator used in the synthesis left in the solution [44], which may explain the low yield of product **2d** (Table 1, Entry 17). The Mitsunobu reaction between purine **1a** and cyclopenten-2-ol was surprisingly slow, and full conversion was not achieved even after several days. Furthermore, the *N*-9/*N*-7 selectivity was only *ca.* 4:1 (Table 1, Entry 18). Pd-catalyzed allylic alkylation of purine **1a** went to completion and gave the isomers **2d** and **3d** in a 4:1 ratio (Table 1, Entry 19).

The 6-chloropurines **2a**, **2b**, and **2c** were readily brominated on *C*-8 simply by treatment of bromine in water (Scheme 1; Table 2; Entries 1, 4, and 5). For compound **2d**, which has an alkene function, the bromide was introduced by *C*-8 lithiation followed by trapping with CCl_2_BrCCl_2_Br (Table 2, Entry 6) [9,32,45,46]. However, the yield was surprisingly low and also another route to bromide **4d** was examined (see below). Finally hydrolysis of the dihalopurines **4**, employing conditions used for hydrolysis of other 8-bromopurines [13,14,15,16,47], gave the 8-oxoguanines **5**. Complete conversion was achieved in the hydrolysis compound **4a**, whereas small amounts of the partly hydrolyzed chlorides **6** where present after hydrolysis of purines **4b**–**d** even after prolonged reaction times.

Attempts to brominate the *O*-carbamoylguanine **2e** failed (Scheme 1). Treatment with bromine or lithiation followed by trapping with CCl_2_BrCCl_2_Br only resulted in cleavage of the carbamoyl protecting group to give the guanine derivative **7**. When compound **2e** was treated with NBS, no reaction took place at all. Thus, no attempts were made to brominate the carbamoyl protected guanines **2f** and **2g**.

Since bromination of the cyclopentenylpurine **2d** turned out to be a challenge, we also examined the possibility for introducing the 8-halo substituent before the *N*-9 alkyl group (Scheme 1). We chose to brominate the THP protected compound **8** [30] and removed the protection group under mild acidic condition, but direct bromination of purine **1a** in a moderate yield has also been reported [48].

Alkylation of 8-bromo-6-chloropurin-2-amine (**10**) by bromomethylcyclohexane in the presence of K_2_CO_3_/DMF (Table 2, Entry 2) occurred slowly compared to alkylation of 2-amino-6-chloropurine (**1a**) under the same set of reaction conditions (for alkylation of compound **1a** see Table 1). NMR analysis showed that approximately 50% of the starting material was intact even after 96 h reaction time and the desired product was isolated in a low yield. Also, *ca.* 4% of *N*-7 alkylated isomer was formed, as judged by NMR. When compound **10** was reacted under Mitsunobu (Table 2, Entry 3) conditions, high conversion (*ca.* 95%) and almost full selectivity towards the desired *N*-9 alkylated isomer **4a** was achieved, as judged by ^1^H-NMR. However, the product **4a** was isolated only in 56% due to tedious separation from reduced DIAD. Since compound **10** reacted slower (conventional alkylation) or comparably (Mitsunobu alkylation) to compound **1a**, it was concluded that there were no benefits associated with introducing the bromide before the *N*-alkyl group for the synthesis of 8-bromopurines **4a**–**c**.

Also, synthesis of the 9-cyclopentenylpurine **4d** by *N*-alkylation of compound **10** was examined (Table 2, Entries 7 and 8) since bromination of 2-amino-6-chloro-9-cyclopentenylpurine **2d** turned out to give only a low yield of the desired product. Again, isolation of the desired product from alkylation under Mitsunobu conditions turned out to be troublesome. We tried this Mitsunobu alkylation using the water-soluble azodicarboxylate DMEAD (di-2-methoxyethyl azodicarboxylate) as well as DIAD [49]. Purification of the product was less complicated, but the conversion was low and *ca.* 40% of starting material **10** was recovered. Also, Pd-catalyzed allylation turned out to be a very slow reaction and even after six days only 29% of the desired compound **4d** could be isolated, together with 32% unconverted starting material **10**.

### 2.2. Biology

**Figure 2:**
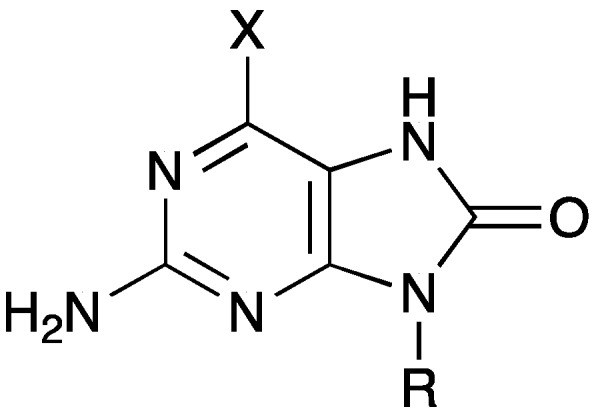
General structure of the compounds shown in Table 3.

As previously mentioned, our hypothesis was that *N*-alkyl-8-oxoguanines may inhibit the human 8-oxoguanine DNA glycosylase (OGG1). Other substituents in the purine 8-position are probably not tolerated, for instance 8-bromo- and 8-aminoguanines are reported to be enhancers for OGG1 activity [50]. Thus, the 8-oxoguanines **5** as well as the partly hydrolyzed 6-chloro-8-oxopurines **6** were tested against human DNA glycosylases OGG1 and NTH1. A general structure of the tested compounds is shown in Figure 2 and the results are presented in Table 3 and Table 4, and Supplementary Figure S19.

**Table 3:** % Activity of OGG1 in the presence of compounds **5** or **6** at 0.2 mM concentration.

Compound	X	R	% Activity
**5a**	OH ^a^	CH_2_-*c*-hexyl	89 ± 5
**5b**	OH ^a^	*c*-hexyl	92 ± 2
**6b**	Cl	*c*-hexyl	70 ± 11
**5c**	OH	*c*-pentyl	101 ± 12
**6c**	Cl	*c*-pentyl	72 ± 9
**5d**	OH	*c*-pent-2-enyl	92 ± 7
**6d**	Cl	*c*-pent-2-enyl	84 ± 3

^a^ The predominant 6-oxo tautomer of compounds **5** is shown in Scheme 1.

**Table 4:** % Activity of NTH1 in the presence of compounds **5** or **6** at 0.5 mM concentration.

Compound	X	R	% Activity
**5a**	OH ^a^	CH_2_-*c*-hexyl	96 ± 3
**5b**	OH ^a^	*c*-hexyl	123 ± 20
**6b**	Cl	*c*-hexyl	73 ± 37
**5c**	OH	*c*-pentyl	102 ± 16
**6c**	Cl	*c*-pentyl	108 ± 18
**5d**	OH	*c*-pent-2-enyl	104 ± 21
**6d**	Cl	*c*-pent-2-enyl	89 ± 13

^a^ The predominant 6-oxo tautomer of compounds **5** is shown in Scheme 1.

Compounds **6b** and **6c** inhibit the OGG1 enzyme by *ca.* 30%, followed by compounds **5a**, **5b**, and **6d** at *ca.* 10%–15%, all at 0.2 mM ligand concentration. Interestingly, the halogenated compounds seem in general to be better inhibitors than their 6-oxo derivatives. To check enzyme specificity, we tested the same seven compounds at the higher concentration of 0.5 mM against NTH1, a structural but not functional homolog of OGG1. Both enzymes have a deep pocket for binding of oxidized bases; in general, OGG1 repairs oxidized purines while NTH1 is involved in repair of oxidized pyrimidines. Compound **6b** reduced the NTH1 activity by around 25% at 0.5 mM ligand concentration. An effect of varying the *N*-9 substituent is not so evident from the few compounds examined.

## 3. Experimental Section

### 3.1. General Information

^1^H-NMR spectra were recorded at 300 MHz with a Bruker DPX 300, at 400 MHz with a Bruker DPX 400 or at 600 with a Bruker AVI 600 instrument (Bruker BioSpin AG, Fällanden, Switzerland). The ^13^C-NMR spectra were recorded at 75, 100, or 150 MHz with the Bruker instruments listed above. Assignments of ^1^H and ^13^C resonances are inferred from 1D ^1^H-NMR, 1D ^13^C-NMR, DEPT, or APT, and 2D NMR (HMQC, HMBC) spectroscopical data. ^1^H- and ^13^C-NMR spectra of all novel compounds can be found in the Supplementary Material (Figures S1–S18). HRMS (EI) was performed with a double-focusing magnetic sector VG Prospec Q instrument (Waters, Manchester, UK) and HRMS (ESI) with a TOF quadrupole Micromass QTOF 2 W instrument (Waters). Melting points were determined with a Büchi Melting point B-545 apparatus (Büchi Labortechnik AG, Flawil, Switzerland) and are uncorrected. Dry DMF and THF were obtained from a solvent purification system, MB SPS-800 (MBraun, Garching, Germany). Acetic anhydride and diisopropylamine were distilled over CaH_2_. DMSO was dried over activated 3 Å molecular sieves for four days. Potassium carbonate was oven dried at 150 °C under high vacuum for 12 h. A saturated aqueous solution of Br_2_ was prepared by stirring water (20 mL) with Br_2_ (0.200 mL) in a closed container for 15 min at ambient temperature. Sodium hydride (*ca.* 60% in mineral oil) was washed with dry pentane under inert atmosphere prior to use. All other reagents were commercially available and used as received. The following compounds were available by literature methods: Cyclohexyl tosylate [51], cyclopentenyl bromide [44], cyclopent-2-enol [52], cyclopentenyl acetate [53], **1b** [29], **8** [30].

### 3.2. Synthesis

#### 3.2.1. 2-Amino-6-chloro-9-(cyclohexylmethyl)-9*H*-purine (**2a**) and 2-Amino-6-chloro-7-(cyclohexylmethyl)-7*H*-purine (**3a**)

*Method A*: K_2_CO_3_ (1.63 g, 11.8 mmol) was added to a stirring solution of compound **1a** (1.00 g, 5.90 mmol) in dry DMF (30 mL) at ambient temperature under N_2_. After 20 min bromomethylcyclohexane (0.905 mL, 6.49 mmol) was added and the resulting mixture was stirred for 72 h, filtered. and evaporated. The isomers were separated by flash chromatography on silica gel, eluting with MeOH–CH_2_Cl_2_ (1:9) to yield **2a** (1.05 g, 67%) and **3a** (150 mg, 10%).

**2a**: colorless solid; mp 148–150 °C (lit. [54], 154–155 °C); ^1^H-NMR (DMSO-*d*_6_, 400 MHz) δ 8.10 (s, 1H, H-8), 6.91 (s, 2H, NH_2_), 3.88 (d, *J* = 7.4 Hz, 2H, NCH_2_), 1.88–1.72 (m, 1H, H-1 in *c*-hex), 1.68–1.52 (m, 3H, *c*-hex), 1.51–1.42 (m, 2H, *c*-hex), 1.19–1.02 (m, 3H, *c*-hex) 1.00–0.85 (m, 2H, *c*-hex); ^13^C-NMR (DMSO-*d*_6_, 100 MHz) δ 159.8 (C, C-2), 154.3 (C, C-4), 149.3 (C, C-6), 143.7 (CH, C-8), 123.3 (C, C-5), 48.8 (CH_2_, NCH_2_), 37.1 (CH, C-1 in *c*-hex), 29.9 (CH_2_, C-3 and C-5 in *c*-hex), 25.8 (CH_2_, C-4 in *c*-hex), 25.0 (CH_2_, C-2 and C-6 in *c*-hex); HREIMS *m*/*z* 265.1092 (calcd for C_12_H_16_ClN_5_, 265.1094). Spectral data were in good agreement with those reported before [54].

**3a**: colorless solid mp 228–231 °C. ^1^H-NMR (DMSO-*d*_6_, 400 MHz) δ 8.32 (s, 1H, H-8), 6.62 (s, 2H, NH_2_), 4.10 (d, *J* = 7.2 Hz, 2H, NCH_2_), 1.82–1.70 (m, 1H, H-1 in *c*-hex), 1.69–1.54 (m, 3H, *c*-hex), 1.50–1.41 (m, 2H, *c*-hex), 1.24–1.06 (m, 3H, *c*-hex), 1.03–0.89 (m, 2H, *c*-hex); ^13^C-NMR (DMSO-*d*_6_, 100 MHz) δ 164.2 (C, C-4), 159.9 (C, C-2), 149.8 (CH, C-8), 142.3 (C, C-6), 114.9 (C, C-5), 51.8 (CH_2_, NCH_2_), 38.6 (CH, C-1 in *c*-hex), 29.6 (CH_2_, C-3 and C-5 in *c*-hex), 25.8 (CH_2_, C-4 in *c*-hex), 25.1 (CH_2_, C-2 and C-3 in *c*-hex); HREIMS *m*/*z* 265.1096 (calcd for C_12_H_16_ClN_5_, 265.1094).

*Method B*: Compound **1a** (200 mg, 1.18 mmol) was added to a solution of cyclohexylmethanol (141 mg, 1.24 mmol) and PPh_3_ (325 mg, 1.24 mmol) in dry THF (10 mL) under N_2_. The resulting suspension was treated with diisopropyl azodicarboxylate (DIAD) (0.244 mL, 1.24 mmol) and the reaction mixture was stirred at 70 °C for 7 h before cyclohexylmethanol (141 mg, 1.24 mmol), PPh_3_ (325 mg, 1.24 mmol), and DIAD (0.244 mL, 1.24 mmol) were added. The mixture was stirred for another 7 h at 70 °C, cooled, treated with brine (10 mL), and extracted with CH_2_Cl_2_ (3 × 75 mL). The combined organic layers were washed with water (50 mL), dried (Na_2_SO_4_) and evaporated *in vacuo.* The isomers were separated by flash chromatography on silica gel eluting with EtOAc–Hexane (gradient; 70%–100% EtOAc) followed by MeOH–EtOAc (1:9) to yield **2a** (240 mg, 76%) and **3a** (16 mg, 5%).

#### 3.2.2. 2-Amino-6-chloro-9-(cyclohexyl)-9*H*-purine (**2b**)

*Method A*: The title compound was prepared from compound **1a** (200 mg, 1.18 mmol), K_2_CO_3_ (326 mg, 2.36 mmol) and cyclohexyl tosylate (450 mg, 1.77 mmol) in DMF (15 mL) as described for the synthesis of compounds **2a** above. MeOH–EtOAc (1:19) was used for flash chromatography to yield **2b** (98 mg, 33%). Colorless needles; mp 163–165 °C (lit. [55], 165 °C); ^1^H-NMR (DMSO-*d*_6_, 400 MHz) δ 8.23 (s, 1H, H-8), 6.88 (s, 2H, NH_2_), 4.28–4.12 (m, 1H, H-1 in *c*-hex), 2.01–1.75 (m, 7H, *c*-hex), 1.45–1.17 (m, 3H, *c*-hex); ^13^C-NMR (DMSO-*d*_6_, 100 MHz) δ 159.5 (C, C-6), 153.5 (C, C-4), 149.3 (C, C-2), 141.2 (CH, C-8), 123.5 (C, C-5), 53.5 (CH, C-1 in *c*-hex), 31.9 (CH_2_, *c*-hex), 25.1 (CH_2_, *c*-hex), 24.7 (CH_2_, *c*-hex); HREIMS *m*/*z* 251.0934 (calcd for C_11_H_14_ClN_5_, 251.0938). Spectral data were in good agreement with those reported before [55].

*Method B*: The title compound was prepared from compound **1a** (1.00 g, 5.90 mmol), cyclohexanol [2 × (620 mg, 6.19 mmol)], PPh_3_ [2 × (1.62 g, 6.19 mmol)] and DIAD [2 × (1.22 mL, 6.19 mmol] in THF (50 mL) as described for the synthesis of compounds **2a** above. After each addition of cyclohexanol the mixture was subjected to sonication for 20 min using a sonicator probe. EtOAc–Hexane (gradient; 30%–100% EtOAc) was used for flash chromatography to yield **2b** (295 mg, 20%).

#### 3.2.3. 2-Amino-6-chloro-9-(cyclopentyl)-9*H*-purine (**2c**) and 2-Amino-6-chloro-7-(cyclopentyl)-7*H*-purine (**3c**)

*Method A*: The title compounds were prepared from compound **1a** (1.00 g, 5.90 mmol), K_2_CO_3_ (1.63 g, 11.8 mmol) and bromocyclopentane (0.696 mL, 6.49 mmol) in DMF (50 mL) as described for the synthesis of compounds **2a** and **3a** above. MeOH–EtOAc (1:19) was used for flash chromatography to yield **2c** (994 mg, 71%) and **3c** (75 mg, 5%).

**2c**: colorless solid; mp 137–140 °C (lit. [55], 142 °C); ^1^H-NMR (DMSO-*d*_6_, 400 MHz) δ 8.20 (s, 1H, H-8), 6.86 (s, 2H, NH_2_), 4.77–4.65 (m, 1H, H-1 in *c*-pent), 2.16–2.02 (m, 2H, *c*-pent) 2.00–1.75 (m, 4H, *c*-pent), 1.72–1.60 (m, 2H, *c*-pent); ^13^C-NMR (DMSO-*d*_6_, 100 MHz) δ 159.3 (C, C-2), 153.7 (C, C-4), 149.1 (C, C-6), 141.4 (CH, C-8), 123.5 (C, C-5), 55.1 (CH, C-1 in *c*-pent), 31.4 (CH_2_, C-3 and C-4 in *c*-pent), 23.2 (CH_2_,C-2 and C-5 in *c*-pent); HREIMS *m*/*z* 237.0777 (calcd for C_10_H_12_ClN_5_, 237.0781). Spectral data were in good agreement with those reported before [34,55,56].

**3c**: colorless solid; mp >230 °C (dec.); ^1^H-NMR (DMSO-*d*_6_, 400 MHz) δ 8.46 (s, 1H, H-8), 6.59 (s, 2H, NH_2_), 5.11–5.01 (m, 1H, H-1 in *c*-pent), 2.24–2.10 (m, 2H, *c*-pent), 2.02–1.90 (m, 2H, *c*-pent), 1.86–1.62 (m, 4H, *c*-pent); ^13^C-NMR (DMSO-*d*_6_, 100 MHz) δ 164.3 (C, C-4), 159.7 (C, C-2), 146.6 (CH, C-8), 142.2 (C, C-6), 115.1 (C, C-5), 58.0 (CH, C-1 in *c*-pent), 32.6 (CH_2_, C-3 and C-4 in *c*-pent), 23.1 (CH_2_, C-2 and C-5 in *c*-pent); HREIMS *m*/*z* 237.0776 (calcd for C_10_H_12_ClN_5_, 237.0781). Spectral data were in good agreement with those reported before [34,55].

*Method B*: The title compounds were prepared from compound **1a** (200 mg, 1.18 mmol), cyclopentanol [2 × (107 mg, 1.24 mmol)], PPh_3_ [2 × (325 mg, 1.24 mmol)] and DIAD [2 × (244 µL, 1.24 mmol)] in THF (10 mL) as described for the synthesis of compounds **2a** and **3a** above. EtOAc–hexane (gradient; 70%–100% EtOAc) followed by MeOH–EtOAc (1:9) were used for flash chromatography to **2c** (202 mg, 72%) and **3c** (6 mg, 6%).

#### 3.2.4. 2-Amino-6-chloro-9-(cyclopent-2-enyl)-9*H*-purine (**2d**) and 2-Amino-6-chloro-7-(cyclopent-2-enyl)-7*H*-purine (**3d**)

*Method A*: The title compound **2d** was prepared from compound **1a** (200 mg, 1.18 mmol), K_2_CO_3_ (490 mg, 3.54 mmol) and 3-bromocyclopentene (0.29 mL, *ca.* 80% pure, *ca.* 2.4 mmol) in DMF (20 mL) as described for the synthesis of compounds **2a** and **3a** above, except that the reaction time was 24 h. EtOAc–hexane (gradient; 50%–100% EtOAc) followed by MeOH–EtOAc (1:9) were used for flash chromatography to yield **2d** (49 mg, 18%). Colorless solid; mp 154–154.5 °C (lit., [57] 166.0–166.7 °C); ^1^H-NMR (DMSO-*d*_6_, 400 MHz) δ 7.96 (s, 1H, H-8), 6.88 (s, 2H, NH_2_), 6.26–6.18 (m, 1H, *c*-pent), 5.93–5.84 (m, 1H, *c*-pent), 5.51–5.41 (m, 1H, *c*-pent), 2.73–2.61 (m, 1H, *c*-pent), 2.47–2.36 (m, 2H, *c*-pent), 2.00–1.87 (m, 1H, *c*-pent); ^13^C-NMR (DMSO-*d*_6_, 100 MHz) δ 159.6 (C, C-6), 153.6 (C, C-4), 149.3 (C, C-2), 141.1 (CH, C-8), 137.3 (CH, C-2 in *c*-pent), 128.6 (CH, C-3 in *c*-pent), 123.6 (C, C-5), 59.4 (CH, C-1 in *c*-pent), 31.2 (CH_2_, C-5 in *c*-pent), 30.4 (CH_2_, C-4 in *c*-pent); HREIMS *m*/*z* 235.0624 (calcd for C_10_H_10_ClN_5_ 235.0625). Spectral data were in good agreement with those reported before [57].

*Method B*: The title compound **2d** was prepared from compound **1a** (340 mg, 2.01 mmol), cyclopent-2-enol [2 × (0.180 mL, 2.03 mmol)], PPh_3_ [2 × (531 mg, 2.03 mmol)] and DIAD [2 × (0.442 mL, 2.03 mmol)] in THF (20 mL) as described for the synthesis of compounds **2a** and **3a** above. EtOAc–Hexane (gradient; 70%–100% EtOAc) followed by MeOH–EtOAc (1:9) were used for flash chromatography to yield **2d** (187 mg, 40%).

*Method C*: A solution of compound **1a** (100 mg, 0.590 mmol) and NaH (18 mg, 0.77 mmol) in dry DMSO (5 mL) was stirred at room temperature for 20 min under Ar atmosphere. The mixture was added to a solution of cyclopent-2-e*n*-1-yl acetate (0.070 mL, 0.77 mmol) and Pd(PPh_3_)_4_ (103 mg, 0.0890 mmol) in dry DMSO (5 mL) and the resulting mixture was stirred at 50 °C for 48 h under Ar and evaporated *in vacuo.* The product was purified by flash chromatography as described in Method B to yield **2d** (73 mg, 53%) and **3d** (25 mg, 18%).

**3d**: colorless solid; mp 155–157 °C (dec.); ^1^H-NMR (DMSO-*d*_6_, 400 MHz) δ 8.15 (s, 1H, H-8), 6.61 (s, 2H, NH_2_), 6.34–6.28 (m, 1H, *c*-pent), 6.03–5.96 (m, 1H, *c*-pent), 5.82–5.75 (m, 1H, *c*-pent) 2.61–2.34 (m, 3H, *c*-pent) 1.96–1.83 (m, 1H, *c*-pent); ^13^C-NMR (DMSO-*d*_6_, 100 MHz) δ 164.4 (C, C-4), 159.8 (C, C-2), 146.2 (CH, C-8), 142.3 (C, C-6), 138.3 (CH, C-2 in *c*-pent), 127.9 (CH, C-3 in *c*-pent), 114.8 (C, C-5), 62.5 (CH, C-1 in *c*-pent), 32.1 (CH_2_, C-5 in *c*-pent), 31.0 (CH_2_, C-4 in *c*-pent); HREIMS *m*/*z* 235.0631 (calcd for C_10_H_10_ClN_5_, 235.0625). Spectral data were in good agreement with those reported before [57].

#### 3.2.5. 2-Acetamido-9-(cyclohexylmethyl)-9*H*-purin-6-yl diphenylcarbamate (**2e**) and 2-Acetamido-7-(cyclohexylmethyl)-7*H*-purin-6-yl diphenylcarbamate (**3e**)

*Method A*: The title compounds were prepared from compound **1b** (200 mg, 0.515 mmol), K_2_CO_3_ (142 mg, 1.03 mmol) and bromomethylcyclohexane (0.144 mL, 1.03 mmol) in DMF (7 mL) as described for the synthesis of compounds **2a** and **3a** above. MeOH–CH_2_Cl_2_ (1:9) followed by MeOH–CH_2_Cl_2_ (1:4) were used for flash chromatography to yield **2e** (111 mg, 45%) and **3e** (18 mg, 7%).

**2e**: colorless solid; mp. 192–194 °C; ^1^H-NMR (CDCl_3_, 400 MHz) δ 7.97 (s, 1H, NH), 7.87 (s, 1H, H-8), 7.47–7.24 (m, 10H, Ph), 3.99 (d, *J* = 7.0 Hz, 2H, NCH_2_), 2.58 (s, 3H, CH_3_), 1.85 (m, 1H, H-1 in *c*-hex), 1.78–1.58 (m, 5H, *c*-hex), 1.30–1.07 (m, 3H, *c*-hex), 1.07–0.92 (m, 2H, *c*-hex); ^13^C-NMR (CDCl_3_, 100 MHz) δ 171.1 (C, CONH), 156.2 (C, OCON), 155.4 (C, C-4), 152.2 (C, C-2), 150.6 (C, C-6), 144.4 (CH, C-8), 141.9 (C, Ph), 129.3 (CH, Ph), 127.2 (br, 2 × CH_2_, Ph), 120.6 (C, C-5), 50.5 (CH_2_, NCH_2_), 38.4 (CH, C-1 in *c*-hex), 30.8 (CH_2_, C-3 and C-5 in *c*-hex), 26.1 (CH_2_, C-4 in *c*-hex), 25.6 (CH_2_, C-2 and C-3 in *c*-hex), 25.3 (CH_3_); HRESIMS *m*/*z* 485.2311 (calcd for C_27_H_29_N_6_O_3_ + 1, 485.2301).

**3e**: colorless oil; ^1^H-NMR (CDCl_3_, 400 MHz) δ 8.10 (s, 1H, NH), 7.96 (s, 1H, H-8), 7.42–7.36 (m, 8H, Ph), 7.33–7.28 (m, 2H, Ph), 3.89 (d, *J* = 7.2 Hz, 2H, NCH_2_), 2.63 (s, 3H, CH_3_), 1.68–1.60 (m, 3H, *c*-hex), 1.45–1.35 (m, 2H, *c*-hex), 1.13–0.98 (m, 3H, *c*-hex), 0.92–0.72 (m, 3H, *c*-hex); ^13^C-NMR (CDCl_3_, 100 MHz) δ 172.0 (C, CONH), 164.9 (C-4), 152.2 (C, C-2), 151.9 (C, OCON), 149.5 (C, C-6), 148.5 (CH, C-8), 141.5 (C, Ph), 129.6 (CH, Ph), 127.6 (br, 2 × CH, Ph), 111.9 (C, C-5), 53.8 (CH_2_, NCH_2_), 38.9 (CH, C-1 in *c*-hex), 30.3 (CH_2_, C-3 and C-5 in *c*-hex), 26.0 (CH_2_,C-4 in *c*-hex) 25.4 (CH_2_, C-2 and C-6 in *c*-hex ), 25.6 (CH_3_); HRESIMS *m*/*z* 485.2313 (calcd for C_27_H_29_N_6_O_3_ + 1, 485.2301).

*Method B*: The title compounds were prepared from compound **1b** (200 mg, 0.515 mmol), cyclohexylmethanol [2 × (62 mg, 0.54 mmol)], PPh_3_ [2 × (142 mg, 0.540 mmol)] and DIAD [2 × (0.106 mL, 0.540 mmol)] in THF (10 mL) as described for the synthesis of compounds **2a** and **3a** above. EtOAc–Hexane (gradient; 70%–100% EtOAc) followed by MeOH–EtOAc (1:9) were used for flash chromatography to yield **2e** (175 mg, 70%) and **3e** (7 mg, 3%).

#### 3.2.6. 2-Acetamido-9-(cyclohexyl)-9*H*-purin-6-yl diphenylcarbamate (**2f**)

*Method A*: The title compound was prepared from compound **1b** (500 mg, 1.29 mmol), K_2_CO_3_ (329 mg, 2.38 mmol) and cyclohexyl tosylate (441 mg, 1.73 mmol) in THF (15 mL) as described for the synthesis of compounds **2a** above. MeOH–EtOAc (1:19) was used for flash chromatography to yield **2f** (180 mg, 30%). Off-white solid; mp 189–190 °C; ^1^H-NMR (DMSO-*d*_6_, 300 MHz) δ 10.67 (s, 1H, NH), 8.55 (s, 1H, H-8), 7.54–7.38 (m, 8H, Ph), 7.37–7.25 (m, 2H), 4.46–4.28 (m, 1H, H-1 in *c*-hex), 2.20 (s, 3H, CH_3_), 2.06–1.80 (m, 6H, CH in *c*-hex), 1.76–1.65 (m, 1H, *c*-hex), 1.51–1.14 (m, 3H, *c*-hex); ^13^C-NMR (DMSO-*d*_6_, 75 MHz) δ 168.8 (C, CONH), 155.0 (C, OCON), 154.3 (C, C-4), 151.7 (C, C-2), 150.3 (C, C-6), 144.1 (CH, C-8), 141.6 (C, Ph), 129.4 (CH, Ph), 127.1 (CH, Ph), 120.1 (C, C-5), 54.1 (CH, C-1 in *c*-hex), 31.9 (CH_2_, C-3 and C-5 in *c*-hex), 25.1 (CH_2_, C-2 and C-6 in *c*-hex), 24.7 (CH_2_, C-4 in *c*-hex), 24.6 (CH_3_); HREIMS *m*/*z* 470.2057 (calcd for C_26_H_26_N_6_O_3_, 470.2066). 

*Method B*: The title compound was prepared from compound **1b** (400 mg, 1.03 mmol), cyclohexanol [2 × (108 mg, 1.08 mmol)], PPh_3_ [2 × (284 mg, 1.08 mmol)] and DIAD [2 × (0.213 mL, 1.08 mmol)] in THF (10 mL) as described for the synthesis of compound **2a** above. EtOAc–Hexane (gradient; 30%–100% EtOAc) was used for flash chromatography to yield **2f** (107 mg, 22%) as an off-white solid.

#### 3.2.7. 2-Acetamido-9-(cyclopentyl)-9*H*-purin-6-yl diphenylcarbamate (**2g**)

*Method A*: The title compound **2g** was prepared from compound **1b** (389 mg, 1.00 mmol), K_2_CO_3_ (277 mg, 2.00 mmol) and bromocyclopentane (0.120 mL, 1.10 mmol) in DMF (50 mL) as described for the synthesis of compounds **2a** above. MeOH–EtOAc (1:19) was used for flash chromatography to yield **2g** (238 mg, 52%). Colorless solid; mp 137–140 °C; ^1^H-NMR (DMSO-*d*_6_, 400 MHz) δ 10.62 (s, 1H, NH), 8.51 (s, 1H, H-8), 7.53–7.40 (m, 8H, Ph), 7.36–7.27 (m, 2H, Ph), 4.79–4.62 (m, 1H, H-1 in *c*-pent), 2.21 (s, 3H, CH_3_), 2.19–2.11 (m, 2H, *c*-pent), 2.10–1.83 (m, 4H, *c*-pent), 1.77–1.62 (m, 2H, *c*-pent); ^13^C-NMR (DMSO-*d*_6_, 100 MHz) δ 168.8 (C, CONH), 155.0 (C, OCON), 154.6 (C, C-4), 151.7 (C, C-2), 150.2 (C, C-6), 144.4 (CH, C-8), 141.6 (C, Ph), 129.4 (CH, Ph), 127.2 (CH, Ph), 120.3 (C, C-5), 56.1 (CH, C-1 in *c*-pent), 31.7 (CH_2_, C-3 and C-4 in *c*-pent), 24.5 (CH_3_), 23.5 (CH_2_, C-2 and C-5 in *c*-pent), one Ph signal was hidden; HREIMS *m*/*z* 456.1903 (calcd for C_25_H_24_N_6_O_3_, 456.1910).

*Method B*: The title compound **2g** was prepared from compound **1b** (389 mg, 1.00 mmol), cyclopentanol [2 × (91 mg, 1.1 mmol)], PPh_3_ [2 × (276 mg, 1.05 mmol)] and DIAD [2 × (0.207 mL, 1.05 mmol)] in THF (10 mL) as described for the synthesis of compounds **2a** above. EtOAc–Hexane (gradient; 70%–100% EtOAc) followed by MeOH–EtOAc (1:9) were used for flash chromatography to yield **2g** (264 mg, 58%).

#### 3.2.8. 2-Amino-8-bromo-6-chloro-9-(cyclohexylmethyl)-9*H*-purine (**4a**)

*Method A*: Sat. aq. Br_2_ (50 mL) was added dropwise to a rapidly stirred suspension of **2a** (1.50 g, 5.64 mmol) in water (20 mL) over 10 min at ambient temperature. The flask was closed and the reaction mixture was stirred for 74 h. The flask was left open in the hood until all Br_2_ was evaporated before the water was removed *in vacuo*. The product was purified by flash chromatography on silica gel, eluting with EtOAc–Hexane (1:1) to yield **4a** (1.55 g, 79%). Yellow solid; mp 169–170 °C. ^1^H-NMR (DMSO-*d*_6_, 400 MHz) δ 7.07 (s, 2H, NH_2_), 3.86 (d, *J* = 8.0 Hz, 2H, NCH_2_), 1.96–1.81 (m, 1H, H-1 in *c*-hex), 1.72–1.44 (m, 5H, *c*-hex), 1.24–0.92 (m, 5H, *c*-hex); ^13^C-NMR (DMSO-*d*_6_, 100 MHz) δ 159.7 (C, C-2), 155.0 (C, C-4), 148.0 (C, C-6), 129.5 (C, C-8), 123.3 (C, C-5), 49.6 (CH_2_, NCH_2_), 36.9 (CH, *c*-hex), 30.0 (CH_2_, *c*-hex), 25.7 (CH_2_, *c*-hex), 25.1 (CH_2_, *c*-hex); HREIMS *m*/*z* 343.0198 (calcd for C_12_H_15_BrClN_5_, 343.0199).

*Method B*: K_2_CO_3_ (231 mg, 1.67 mmol) was added to a stirring solution of compound **10** (207 mg, 0.833 mmol) in dry DMF (15 mL) at ambient temperature under N_2_. After 20 min, bromomethylcyclohexane (0.175 mL, 1.25 mmol) was added and the resulting mixture was stirred for 80 h before K_2_CO_3_ (115 mg, 0.835 mmol) and bromomethylcyclohexane (0.175 mL, 1.25 mmol) was added and the mixture was stirred for additional 16 h and evaporated *in vacuo.* The product was purified by flash chromatography on silica gel eluting with EtOAc–Hexane (2:3) to yield **4a** (98 mg, 34%).

*Method C*: The title compound was prepared from compound **10** (175 mg, 0.704 mmol), cyclohexylmethanol [2 × (0.091 mL, 0.74 mmol)], PPh_3_ [2 × (276 mg, 0.740 mmol)] and DIAD [2 × (0.146 mL, 0.740 mmol)] in THF (10 mL) as described for the synthesis of compounds **2a** above. EtOAc–Hexane (gradient; 20%–100% EtOAc) was used for flash chromatography to yield **4a** (136 mg, 56%).

#### 3.2.9. 2-Amino-8-bromo-6-chloro-9-(cyclohexyl)-9*H*-purine (**4b**)

The title compound was prepared from compound **2b** (250 mg, 0.993 mmol) and saturated aqueous Br_2_ (12 mL) in water (5 mL) as described for the synthesis of compound **4a** above. EtOAc–Hexane (1:1) was used for flash chromatography to yield **4b** (229 mg, 70%). Yellow solid; mp 181–183 °C; ^1^H-NMR (DMSO-*d*_6_, 600 MHz) δ 6.98 (br s, 2H, NH_2_), 4.35–4.22 (m, 1H, H-1 in *c*-hex), 2.46–2.27 (m, 2H, *c*-hex), 1.92–1.75 (m, 4H, *c*-hex), 1.74–1.62 (m, 1H, *c*-hex), 1.45–1.29 (m, 2H, *c*-hex), 1.28–1.12 (m, 1H, *c*-hex); ^13^C-NMR (DMSO-*d*_6_, 150 MHz) δ 159.1 (C, C-2), 154.5 (C, C-4), 148.2 (C, C-6), 128.6 (C, C-8), 123.7 (C, C-5), 57.6 (CH, C-1 in *c*-hex), 29.7 (CH_2_, C-3 and C-5 in *c*-hex), 25.3 (CH_2_, C-2 and C-6 in *c*-hex), 24.6 (CH_2_, C-4 in *c*-hex); HRESIMS *m*/*z* 330.0131 (calcd for C_11_H_14_BrClN_5_ + 1, 330.0121).

#### 3.2.10. 2-Amino-8-bromo-6-chloro-9-(cyclopentyl)-9*H*-purine (**4c**)

The title compound was prepared from compound **2c** (880 mg, 3.70 mmol) and sat. aq. Br_2_ (35 mL) in water (10 mL) as described for the synthesis of compound **4a** above. EtOAc–Hexane (1:1) was used for flash chromatography to yield **4c** (950 mg, 81%).Yellow solid; mp 172–174 °C; ^1^H-NMR (DMSO-*d*_6_, 600 MHz) δ 6.97 (s, 2H, NH_2_), 4.85–4.77 (m, 1H, H-1 in *c*-pent), 2.33–2.17 (m, 2H, *c*-pent), 2.11–1.87 (m, 4H, *c*-pent), 1.71–1.59 (m, 2H, *c*-pent); ^13^C-NMR (DMSO-*d*_6_, 150 MHz) δ 159.1 (C, C-2), 154.3 (C, C-4), 148.2 (C, C-6), 129.3 (C, C-8), 123.9 (C, C-5), 57.7 (CH, C-1 in *c*-pent), 29.7 (CH_2_, C-3 and C-4 in *c*-pent), 24.4 (CH_2_, C-2 and C-5 in *c*-pent); HREIMS *m*/*z* 314.9880 (calcd for C_10_H_11_BrClN_5_, 314.9886).

#### 3.2.11. 2-Amino-8-bromo-6-chloro-9-(cyclopent-2-enyl)-9*H*-purine (**4d**)

*Method A*: A solution of diisopropylamine (0.145 mL, 1.03 mmol) in dry THF (3 mL) was stirred at −78 °C under Ar. *n*-BuLi (0.536 mL, 1.00 mmol, 1.87 M in hexane) was added dropwise. After stirring for 30 min, a solution of compound **2d** (118 mg, 0.500 mmol) in THF (1.5 mL) was added. After additional stirring for 1 h at −78 °C, a solution of CBrCl_2_CBrCl_2_ (326 mg, 1.00 mmol) in THF (1.5 mL) was added dropwise and the resulting mixture was stirred at −78 °C for 1 h, and then 10 min without cooling. Saturated aqueous NH_4_Cl (5 mL) was added and the resulting mixture was extracted with EtOAc (3 × 50 mL). The combined organic extracts were washed with brine (100 mL), dried (MgSO_4_), and evaporated *in vacuo*. The product was purified by flash chromatography on silica gel eluting with EtOAc–Hexane (1:1) to yield **4d** (50 mg, 32%). Buff solid; mp 157–158 °C (dec.); ^1^H-NMR (DMSO-*d*_6_, 600 MHz) δ 6.95 (s, 2H, NH_2_), 6.15–6.13 (m, 1H, *c*-pent), 5.74–5.72 (m, 1H, *c*-pent), 5.69–5.60 (m, 1H, *c*-pent) 2.90–2.79 (m, 1H, *c*-pent), 2.48–2.36 (m, 2H, *c*-pent), 2.22–2.14 (m, 1H, *c*-pent); ^13^C-NMR (DMSO-*d*_6_, 150 MHz) δ 159.3 (C, C-2), 154.4 (C-4), 148.0 (C, C-6), 136.5 (CH, C-3 in *c*-pent), 128.0 (C, C-8), 127.7 (CH, C-2 in *c*-pent), 123.5 (C-5), 62.1 (CH, C-1 in *c*-pent), 32.0 (CH_2_, C-5 in *c*-pent), 27.9 (CH_2_, C-4 in *c*-pent); HREIMS *m*/*z* 312.9734 (calcd for C_10_H_9_BrClN_5_, 312.9730).

*Method B*: Compound **10** (64 mg, 0.26 mmol) was added to a cooled solution of cyclopent-2-en-1-ol (44 mg, 0.51 mmol) and PPh_3_ (135 mg, 0.515 mmol) in anhydrous THF (5 mL) under Ar. The resulting suspension was treated with diethyl azodicarboxylate (DEAD, 0.080 mL, 0.51 mmol) and the resulting mixture was stirred at ambient temperature for 1 h and at 70 °C for 15 h. The mixture was cooled, treated with brine (50 mL), and extracted with CH_2_Cl_2_ (3 × 50 mL). The combined organic layer was washed with water (10 mL), dried (Na_2_SO_4_), and evaporated *in vacuo*. The product was purified by flash chromatography on silica gel eluting with EtOAc–Hexane (3:7) to yield **4d** (34 mg, 42%).

*Method C*: A solution of compound **10** (110 mg, 0.423 mmol) and NaH (16 mg, 0.67 mmol) in dry DMF (10 mL) was stirred at ambient temperature for 20 min under Ar. Pd(PPh_3_)_4_ (77 mg, 0.067 mmol) and cyclopent-2-e*n*-1-yl acetate (84 mg, 0.66 mmol) were added, and the resulting mixture was stirred at 55 °C. After three days Pd(PPh_3_)_4_ (77 mg, 0.067 mmol) and cyclopent-2-e*n*-1-yl acetate (84 mg, 0.66 mmol) were added. The reaction mixture was stirred for three more days and evaporated under *in vacuo*. The product was purified by flash chromatography on silica gel eluting with EtOAc–Hexane (gradient 50%–100% EtOAc) followed by MeOH–EtOAc (1:9) to yield **4d** (41 mg, 29%).

#### 3.2.12. 9-(Cyclohexylmethyl)-8-oxoguanine (**5a**)

A mixture of compound **4a** (263 mg, 0.763 mmol), NaOAc (319 mg, 3.89 mmol), glacial AcOH (9 mL), and Ac_2_O (1.5 mL, 17 mmol) was stirred at reflux under N_2_ for 16 h, before the mixture was cooled and evaporated *in vacuo*. The residue was suspended in water (3 mL) and stirred at ambient temperature while the pH was adjusted to 13 by dropwise addition of 10M NaOH (aq). The resulting solution was refluxed for 20 min, cooled to 0 °C, and stirred while the pH was brought down to 7 by dropwise addition of 6M HCl (aqueous). The precipitate was collected and dried *in vacuo*. The product was purified by flash chromatography on silica gel eluting with MeOH–CH_2_Cl_2_ (1:4) to yield **5a** (160 mg, 80%). Pinkish solid; mp 297–300 °C; ^1^H-NMR (DMSO-*d*_6_, 400 MHz) δ 10.57 (s, 1H, NH), 10.48 (s, 1H, NH), 6.43 (s, 2H, NH_2_), 3.42 (d, *J* = 7.4 Hz, 2H, NCH_2_), 1.85–1.71 (m, 1H, H-1 in *c*-hex), 1.69–1.48 (m, 5H, *c*-hex), 1.19–1.06 (m, 3H, *c*-hex), 0.98–0.85 (m, 2H, *c*-hex); ^13^C-NMR (DMSO-*d*_6_, 100 MHz) δ 153.4 (C, C-6), 152.6 (C, C-8), 150.9 (C, C-2), 148.2 (C, C-4), 98.0 (C, C-5), 44.9 (CH_2_, NCH_2_), 36.3 (CH, C-1 in *c*-hex), 30.1 (CH_2_, C-3 and C-5 in in *c*-hex), 25.9 (CH_2_, C-4 in *c*-hex), 25.1 (CH_2_, C-2 and C-6 in *c*-hex); HREIMS *m*/*z* 263.1380 (calcd for C_12_H_17_N_5_O_2_, 263.1382).

#### 3.2.13. 9-(Cyclohexyl)-8-oxoguanine (**5b**) and 2-Amino-6-chloro-9-cyclohexyl-7*H*-purin-8(9*H*)-one (**6b**)

The title compounds were prepared from compound **4b** (186 mg, 0.563 mmol), NaOAc (231 mg, 2.81 mmol), glacial AcOH (7 mL), and Ac_2_O (1.20 mL, 12.7 mmol) as described for the synthesis of compound **5a** above, except that the reflux time with NaOH was 4 h. MeOH–EtOAc (1:9) was used for flash chromatography to yield **5b** (106 mg, 76%) and **6b** (7 mg, 11%).

**5b**: colorless solid; mp 367–368 °C; ^1^H-NMR (DMSO-*d*_6_, 400 MHz) δ 10.57 (s, 1H, NH), 10.45 (s, 1H, NH), 6.37 (s, 2H, NH_2_), 4.04–3.91 (m, 1H, H-1 in *c*-hex), 2.28–2.11 (m, 2H, *c*-hex), 1.86–1.71 (m, 2H, *c*-hex), 1.69–1.52 (m, 3H, *c*-hex), 1.36–1.04 (m, 3H, *c*-hex); ^13^C-NMR (DMSO-*d*_6_, 100 MHz) δ 152.9 (C, C-6), 151.8 (C, C-8), 150.9 (C, C-2), 147.7 (C, C-4), 98.1 (C, C-5), 51.2 (CH, C-1 in *c*-hex), 29.5 (CH_2_, C-3 and C-5 in *c*-hex), 25.5 (CH_2_, C-2 and C-6 in *c*-hex), 24.8 (CH_2_, C-4 in *c*-hex); HREIMS *m*/*z* 249.1222 (calcd for C_11_H_15_N_5_O_2_, 249.1226).

**6b**: yellow solid mp 320–321 °C; ^1^H-NMR (DMSO-*d*_6_, 300 MHz) δ 11.20 (br s, 1H, NH), 6.54 (s, 2H, NH_2_), 4.12–3.98 (m, 1H, H-1 in *c*-hex), 2.27–2.12 (m, 2H, *c*-hex), 1.88–1.75 (m, 2H, *c*-hex), 1.73–1.58 (m, 3H, *c*-hex), 1.39–1.09 (m, 3H, *c*-hex); ^13^C-NMR (DMSO-*d*_6_, 75 MHz) δ 158.3 (C, C-8), 152.5 (C, C-4), 152.3 (C, C-2), 135.5 (C, C-6), 109.8 (C, C-5), 51.7 (CH, C-1 in *c*-hex), 29.1 (CH_2_, C-3 and C-5 in *c*-hex), 25.4 (CH_2_, C-2 and C-6 in *c*-hex), 24.8 (CH_2_,C-4 in *c*-hex); HREIMS *m*/*z* 267.0877 (calcd for C_11_H_14_ClN_5_O, 267.0887).

#### 3.2.14. 9-(Cyclopentyl)-8-oxoguanine (**5c**) and 2-Amino-6-chloro-9-cyclopentyl-7*H*-purin-8(9*H*)-one (**6c**)

The title compounds were prepared from compound **4c** (250 mg, 0.790 mmol), NaOAc (325 mg, 3.96 mmol), glacial AcOH (10 mL), and Ac_2_O (3.00 mL, 31.6 mmol) as described for the synthesis of compound **5a** above, except that the refluxing time with NaOH was 6 h. MeOH–EtOAc (1:9) was used for flash chromatography to yield **5c** (130 mg, 70%) and **6c** (12 mg, 6%).

**5c**: colorless solid; mp 309–310 °C; ^1^H-NMR (DMSO-*d*_6_, 400 MHz) δ 10.58 (s, 1H, NH), 10.47 (s, 1H, NH), 6.35 (s, 2H, NH_2_), 4.46–4.42 (m, 1H, H-1 in *c*-pent), 2.17–2.01 (m, 2H, *c*-pent), 1.94–1.73 (m, 4H, *c*-pent), 1.63–1.50 (m, 2H, *c*-pent); ^13^C-NMR (DMSO-*d*_6_, 100 MHz) δ 152.9 (C, C-6), 151.9 (C, C-8), 150.9 (C, C-2), 147.8 (C, C-4), 98.2 (C, C-5), 51.8 (CH, C-1 in *c*-pent), 29.0 (CH_2_, C-2 and C-5 in *c*-pent), 24.3 (CH_2_, C-3 and C-4 in *c*-pent); HREIMS *m*/*z* 235.1067 (calcd for C_10_H_13_N_5_O_2_, 235.1069).

**6c**: colorless solid; mp 321–322 °C (dec.); ^1^H-NMR (DMSO-*d*_6_, 400 MHz) δ 11.20 (s, 1H, NH), 6.52 (s, 2H, NH_2_), 4.70–4.46 (m, 1H, *c*-pent), 2.20–2.01 (m, 2H, *c*-pent), 1.98–1.74 (m, 4H, *c*-pent), 1.68–1.50 (m, 2H, *c*-pent); ^13^C-NMR (DMSO-*d*_6_, 100 MHz) δ 158.3 (C, C-4), 152.6 (C, C-8), 152.2 (C, C-6), 135.5 (C, C-2), 109.9 (C, C-5), 52.1 (CH, C-1 in *c*-pent), 28.7 (CH_2_, C-2 and C-5 in *c*-pent), 24.3 (CH_2_, C-3 and C-4 in *c*-pent); HREIMS *m*/*z* 253.0727 (calcd for C_10_H_12_ClN_5_O, 253.0734).

#### 3.2.15. 9-(Cyclopent-2-enyl)-8-oxoguanine (**5d**) and 2-Amino-6-chloro-9-(cyclopent-2-enyl)-7*H*-purin-8(9*H*)-one (**6d**)

The title compounds were prepared from compound **4d** (210 mg, 0.668 mmol), NaOAc (274 mg, 3.34 mmol), glacial AcOH (8 mL), and Ac_2_O (2.78 mL, 29.4 mmol) as described for the synthesis of compound **5a** above, except that the refluxing time with NaOH was 30 h and the heating bath was kept at 160 °C in the first reaction step. Glacial AcOH was used for the final neutralization and EtOAc followed by MeOH–EtOAc (1:9) were used for flash chromatography to yield **5d** (119 mg, 71%) and **6d** (9 mg, 5%).

**5d**: colorless solid; mp 322–325 °C (dec.); ^1^H-NMR (DMSO-*d*_6_, 400 MHz) δ 10.60 (s, 1H, NH), 10.49 (s, 1H, NH), 6.34 (s, 2H, NH_2_), 5.99–5.96 (m, 1H, H-3 in *c*-pent), 5.62–5.59 (m, 1H, H-2 in *c*-pent), 5.30–5.21 (m, 1H, H-1 in *c*-pent), 2.79–2.63 (m, 1H, H-5_a_ in *c*-pent), 2.40–2.26 (m, 1H, H-5_b_ in *c*-pent), 2.24–2.13 (m, 1H, H-4_a_ in *c*-pent), 2.13–2.03 (m, 1H, H-4_b_ in *c*-pent); ^13^C-NMR (DMSO-*d*_6_, 100 MHz) δ 153.0 (C, C-6), 151.8 (C, C-8), 151.0 (C, C-2), 147.7 (C, C-4), 134.5 (CH, C-3 in *c*-pent), 129.0 (CH, C-2 in *c*-pent), 98.3 (C, C-5), 56.8 (CH, C-1 in *c*-pent), 31.8 (CH_2_, C-4 in *c*-pent), 27.4 (CH_2_, C-5 in *c*-pent); HREIMS *m*/*z* 233.0914 (calcd for C_10_H_11_N_5_O_2_, 233.0913).

**6d**: yellow solid; mp 310–310.5 °C; ^1^H-NMR (DMSO-*d*_6_, 300 MHz) δ 11.18 (s, 1H, NH), 6.48 (s, 2H, NH_2_), 6.06–6.02 (m, 1H, H-3 in *c*-pent), 5.65–5.61 (m, 1H, H-2 in *c*-pent), 5.37–5.27 (m, 1H, H-1 in *c*-pent), 2.84–2.69 (m, 1H, H-5_a_ in *c*-pent), 2.43–2.05 (m, 3H, *c*-pent); ^13^C-NMR (DMSO-*d*_6_, 75 MHz) δ 158.3 (C, C-8), 152.4 (C, C-4), 152.1 (C, C-2), 135.4 (C, C-6), 135.3 (CH, C-3 in *c*-pent), 128.1 (CH, C-2 in *c*-pent), 109.9 (C, C-5), 57.2 (CH, C-1 in *c*-pent), 31.8 (CH_2_, C-5 in *c*-pent), 27.0 (CH_2_, C-4 in *c*-pent); HREIMS *m*/*z* 251.0568 (calcd for C_10_H_10_ClN_5_O, 251.0574).

#### 3.2.16. *N*-[9-(Cyclohexylmethyl)-6-oxo-6,9-dihydro-1H-purin-2-yl]acetamide (**7**)

*Method A*: Br_2_ (33 mg, 0.21 mmol) was added slowly to a stirred solution of compound **2e** (20 mg, 0.41 mmol) in CHCl_3_ (4 mL) and the resulting mixture was stirred for 6 h at ambient temperature. The reaction mixture was evaporated to dryness and the product was purified by flash chromatography on silica gel eluting with MeOH–EtOAc (1:19) to yield **7** (10 mg, 84%). Off-white solid; mp 271–273 °C (dec.); ^1^H-NMR (DMSO-*d*_6_, 400 MHz) δ 12.01 (s, 1H, N^2^H) 11.63 (s, 1H, NH), 7.95 (s, 1H, H-8), 3.90 (d, *J* = 7.4 Hz, 2H, NCH_2_), 2.17 (s, 3H, CH_3_), 1.88–1.74 (m, 1H, *c*-hex), 1.70–1.54 (m, 3H, *c*-hex), 1.53–1.44 (m, 2H, *c*-hex), 1.21–1.07 (m, 3H, *c*-hex), 1.01–0.87 (m, 2H, *c*-hex); ^13^C-NMR (DMSO-*d*_6_, 100 MHz) δ 173.5 (C, CON^2^), 154.9 (C, C-6), 148.8 (C, C-4), 147.6 (C, C-2), 140.2 (CH, C-8), 120.0 (C, C-5), 48.9 (CH_2_, NCH_2_), 37.4 (CH, C-1 in *c*-hex), 29.9 (CH_2_, C-3 and C-5 in *c*-hex), 25.8 (CH_2_, C-4 in *c*-hex), 25.0 (CH_2_, C-2 and C-6 in *c*-hex), 23.8 (CH_3_); HREIMS *m*/*z* 289.1534 (calcd for C_14_H_19_N_5_O_2_, 289.1539).

*Method B*: The title compound was prepared from compound **2e** (20 mg, 0.41 mmol), diisopropylamine (0.012 mL, 0.83 mmol), *n*-BuLi (0.060 mL, 0.83 mmol, 1.4 M in hexane) and CBrCl_2_CBrCl_2_ (27 mg, 0.83 mmol) in THF (tot. vol. 3 mL) as described for the synthesis of compound **4d** above, except that the reaction was stirred at −78 °C for 2 h after the addition of CBrCl_2_CBrCl_2_. The product was purified by flash chromatography as described above to yield **7** (7 mg, 59%).

#### 3.2.17. 8-Bromo-6-chloro-*N*,9-bis(tetrahydro-2*H*-pyran-2-yl)-9*H*-purin-2-amine (**9**)

The title compound was prepared from compound **8** (1.00 g, 2.96 mmol), diisopropylamine (0.84 mL, 5.9 mmol), *n*-BuLi (4.23 mL, 5.20 mmol, 1.4 M in hexane), and CBrCl_2_CBrCl_2_ (1.93 g, 5.92 mmol) in THF (tot. vol. 30 mL) as described for the synthesis of compound **4d** above, except that the reaction was stirred at −78 °C for 2 h after the addition of CBrCl_2_CBrCl_2_. EtOAc–Hexane (1:1) was used for flash chromatography to yield **9** (987 mg, 80%). Colorless solid; mp 145–147 °C (dec.); ^1^H-NMR (DMSO-*d*_6_, 300 MHz) δ 8.23 (s, 1H, NH), 5.52 (dd, *J* = 11.0, 2.4 Hz, 1H, CH in THP), 5.13–5.02 (m, 1H, CH in THP), 4.10–3.97 (m, 1H, OCH_2_ in THP), 3.89–3.77 (m, 1H, OCH_2_ in THP), 3.69–3.56 (m, 1H, OCH_2_ in THP), 3.49–3.39 (m, 1H, OCH_2_ in THP), 3.14–2.90 (m, 1H, THP) 2.06–1.29 (m, 11H, CH_2_ in THP); ^13^C-NMR (DMSO-*d*_6_, 75 MHz) δ 157.2 (C, C-2), 154.2 (C, C-8), 148.3 (C, C-6), 129.5 (C, C-4), 124.3 (C, C-5), 84.4 (CH, N9-THP), 80.2 (CH, THP), 68.0 (CH_2_, OCH_2_ in THP), 65.7 (CH_2_, OCH_2_ in THP), 30.1, 27.6, 24.9, 24.5, 22.6 and 22.5 (all CH_2_, THP); HREIMS *m*/*z* 415.0417 (calcd for C_15_H_19_BrClN_5_O_2_, 415.0411).

#### 3.2.18. 2-Amino-8-bromo-6-chloro-1*H*-purine (**10**)

A mixture compound **9** (150 mg, 0.360 mmol), 96% EtOH (10 mL) and 9.6 M HCl (0.5 mL), was stirred at ambient temperature for 30 min and neutralized by the addition of solid KHCO_3_. The resulting mixture was evaporated *in vacuo* and the product was isolated by flash chromatography on silica gel eluting with MeOH–CHCl_3_ (1:50:) to yield **10** (80 mg, 90%) as a yellow solid; mp >300 °C (dec.). ^1^H-NMR (DMSO-*d*_6_, 400 MHz) δ 13.65 (s, 1H, NH), 6.88 (s, 2H, NH_2_); ^13^C-NMR (DMSO-*d*_6_, 100 MHz) δ 159.7, 156.2, 147.1, 126.5, 124.0; HREIMS *m*/*z* 246.9257 (calcd for C_5_H_3_BrClN_5_, 246.9260). Spectral data were in good agreement with those reported before [48].

### 3.3. DNA Glycosylase Activity Assay

The enzyme OGG1 (residues12–327) was diluted to the desired concentration (60 pM) using a protein dilution buffer (15% glycerol, 1 mM EDTA, 25 mM HEPES pH 7.9, 1 mM DTT, 0.1 μg/μL BSA). Enzyme, compound **5** or **6** (0.2 mM), and 5′-^32^P end-labeled duplex DNA containing an 8-oxo-G/C base pair were mixed in a 10 μL reaction volume of 50 mM MOPS pH 7.5, 1 mM EDTA, 5% glycerol, and 1 mM DTT. The sequence of the damaged strand in the DNA substrate used is 5′-GCATGCCTGCACGG-8oxoG-CATGGCCAGATCCCCGGGTACCGAG-3′, which was annealed with a complementary strand containing a C opposite 8oxoG. The reactions were incubated for 10 min at 37 °C, followed by addition of 2.5 μL 0.5 M NaOH and incubation for 20 min at 70 °C, in order to stop the reaction and ensure complete strand cleavage. Then 0.5 M HCl/0.25 M MOPS pH 7.5 (2.5 μL) was added to each sample to neutralize the pH. Formamide DNA loading buffer (15 μL) was added to the reaction mixtures and the samples were incubated at 95 °C for 5 min to denature the DNA. The reaction products were analyzed on 20% denaturing urea gels. The gels were transferred to 3M paper and dried at 80 °C for 45 min. The dry gels were placed in a storage phosphor screen overnight, and subsequently scanned on a Typhoon 9410 Variable Mode Image. ImageQuant TL Version 2003.02 (Amersham Biosciences, Piscataway, NJ, USA) was used to analyze the results. For human NTH1, the same procedure was followed, except that the DNA substrate contained a 5-hydroxyuracil/G base pair instead of the 8oxoG/C pair in the OGG1 substrate. The concentration of NTH1 was 3 nM to make sure the activity in the assay was within the linear range. Compounds were screened at 0.5 mM concentration.

## 4. Conclusions

Synthetic routes to 8-oxoguanines have been examined. The best reaction sequence from chloroguanine **1a** to the target compounds was found to be *N*-9 alkylation, C-8 bromination, and finally simultaneous hydrolysis of both halides. Bromination before *N*-alkylation should only be considered in cases where the *N*-substituent is not compatible with bromination conditions, since a bromide in the purine 8-position lowers the reactivity in *N*-alkylations. In most cases, alkylation with an alkyl halide in the presence of a base compared favorably to reactions under Mitsunobu conditions. 2-Amino-6-chloropurine (**1a**) turned out to be a superior guanine precursor compared to the *O*-carbamoylguanine **1b**. The latter did not result in improved *N*-9 selectivity in the alkylation and was not compatible with standard reaction conditions for C-8 bromination.

Enzymatic assays show that partly hydrolyzed 6-chloro-8-oxopurines **6** are somewhat better OGG1 inhibitors than the 8-oxoguanines **5**. However, an inhibitory effect was only observed when using at least 0.2 mM concentration of the compounds, suggesting that the R-group should be extended even further to make more interactions with the enzyme’s substrate recognition pocket. Further, testing of the same compounds at a 2.5-fold higher concentration against human NTH1, which is a structural homolog of OGG1, showed that the synthesized compounds do not inhibit NTH1 at 0.5 mM, except possibly for a weak effect for compound **6b**. To develop these compounds into more potent inhibitors of OGG1, one possibility is to try compounds with more ribose-like R-groups. In the present study, the R-group contains a cyclic hydrocarbon only, and it would also be interesting to replace this with carbocyclic 2′-deoxyribose derivatives, as in antiviral drugs like abacavir and entecavir. In these nucleoside analog drugs, the R-group is not particularly larger than the R-group in our study, but it contains 5′ and/or 3′ hydroxyl groups. Since the structure of the OGG1 enzyme is known [7], molecular modeling will be included in the search for more potent OGG1 inhibitors in the future.

## Supplementary Material

Supplementary materials can be accessed at: http://www.mdpi.com/1420-3049/20/09/15944/s1.

## References

[1] Chabner B.A., Roberts T.G. (2005). Chemotherapy and the war on cancer. Nat. Rev. Cancer.

[2] Madhusudan S., Middleton M.R. (2005). The emerging role of DNA repair proteins as predictive, prognostic and therapeutic targets in cancer. Cancer Treat. Rev..

[3] Helleday T., Petermann E., Lundin C., Hodgson B., Sharma R.A. (2008). DNA repair pathways as targets for cancer therapy. Nat. Rev. Cancer.

[4] Plummer R. (2010). Perspective on the pipeline of drugs being developed with modulation of DNA damage as a target. Clin. Cancer Res..

[5] Pallis A.G., Karamouzis M.W. (2010). DNA repair pathways and their implication in cancer treatment. Cancer Metast. Rev..

[6] Dalhus B., Lærdahl J.K., Backe P.H., Bjørås M. (2009). DNA base repair–recognition and initiation of catalysis. FEMS Microbiol. Rev..

[7] Bruner S.D., Norman D.P.G., Verdine G.L. (2000). Structural basis for recognition and repair of the endogenous mutagen 8-oxoguanine in DNA. Nature.

[8] Siah H.-S.M., Gundersen L.-L., Gørbitz C.H. (2011). NMR and X-ray structural studies on 3-benzyl-8-bromoadenine. J. Heterocycl. Chem..

[9] Siah H.-S.M., Gundersen L.-L. (2013). Synthetic strategies to 9-substituted 8-oxoadenines. Synth. Commun..

[10] Perini F., Tieckelmann H. (1970). Conversion of ureidomalonates and 5-carbalkoxyhydantoins to 5-ureido-4,6-pyrimidinediones. J. Org. Chem..

[11] Brown R., Joseph M., Leigh T., Swain M.L. (1977). Synthesis and reactions of 7,8-dihydro-8-methylpterin and 9-methylguanine 7-oxide. J. Chem. Soc. Perkin.

[12] Müller H., Carell T.A. (2007). Carbocyclic analog of the oxidatively generated DNA lesion spiroiminodihydantoin. Eur. J. Org. Chem..

[13] Robins M.J., Hatfield P.W., Balzarini J., de Clercq E. (1984). Nucleic acid related compounds. 47. Synthesis and biological activities of pyrimidine and purine “acyclic” nucleoside analogs. J. Med. Chem..

[14] Michael M.A., Cottam H.B., Smee D.F., Robins R.K., Kini G.D. (1993). Alkylpurines as immunopotentiating agents. Synthesis and antiviral activity of certain alkylguanines. J. Med. Chem..

[15] Zheng Q.-H., Wang J.-Q., Fei X., Hutchins G.D. (2003). Synthesis of 8-methoxypenciclovir and 8-methoxyganciclovir through methyl triflate, a new potential approach to label penciclovir and ganciclovir with carbon-11. Synthesis.

[16] DeClue M.S., Monnard P.-A., Bailey J.A., Maurer S.E., Collins G.E., Ziock H.-J., Rasmussen S., Boncella J.M. (2009). Nucleobase mediated, photocatalytic vesicle formation from an ester precursor. J. Am. Chem. Soc..

[17] Kaiya T., Ohta M., Kohda K. (1993). Electrophilic amination of imidazole moieties of 9-ethylguanine and 1-methylbenzimidazole derivatives and reactivities of *N*-aminated products. Tetrahedron.

[18] Werbovetz K.A., Macdonald T.L. (1994). On the mechanism of arylamine-DNA adduct formation. Bioorg. Med. Chem. Lett..

[19] Kaiya T., Fujiwara T., Kohda K. (2000). Syntheses and properties of 1-methyl-3-phenylaminobenzimidazolium salts, models of DNA adducts of N7-arylaminodeoxyguanosinium salt. Chem. Res. Toxicol..

[20] Ferenc G., Padar P., Szolomajer J., Kovacs L. (2009). *N*-alkylated guanine derivatives. Curr. Org. Chem..

[21] Kjellberg J., Liljenberg M., Johansson N.G. (1986). Regioselective alkylation of 6-(β-methoxyethoxy)guanine to give the 9-alkylguanine derivative. Tetrahedron Lett..

[22] Kjellberg J., Hagberg C.E., Malm A., Noren J.O., Johansson N.G. (1986). Studies on the Alkylation of Guanine. 2. The synthesis of acyclic guanosine analogs via the precursor 7-methyl-10-oxo-9,10-dihydropyrimido[1,2-*a*]purine. Acta Chem. Scand. B.

[23] Kjellberg J., Johansson N.G. (1986). Regioselective alkylation of guanine via diacyloxyglyoxal-N^2^-acetylguanine adduct to obtain 7-alkylguanine derivatives. Studies on alkylation of guanine I. J. Heterocycl. Chem..

[24] Kjellberg J., Johansson N.G. (1989). Studies on the alkylation of derivatives of guanines. Nucleosides Nucleotides.

[25] Geen G.R., Kincey P.M., Choudary B.M. (1992). Regiospecific Michael additions with 2-aminopurines. Tetrahedron Lett..

[26] Bisacchi G.S., Singh J., Godfrey J.D., Kissick T.P., Mitt T., Malley M.F., di Marco J.D., Gougoutas J.Z., Mueller R.H., Zahler R. (1995). Regioselective coupling of tetraalkylammonium salts of 6-iodo-2-aminopurine to a cyclobutyl triflate: Efficient preparation of homochiral BMS-180,194, a potent antiviral carbocyclic nucleoside. J. Org. Chem..

[27] Geen G.R., Kincey P.M., Spoors P.G. (2001). Regioselective alkylation of guanines using 2-acetoxytetrahydrofurans. Tetrahedron Lett..

[28] Zou R., Robbins M.J. (1987). High-yield regioselective synthesis of 9-glycosyl guanine nucleosides and analogues via coupling with 2-*N*-acetyl-6-*O*-diphenylcarbamoylguanine. Can. J. Chem..

[29] Dalpozzo R., de Nino A., Maiuolo L., Procopio A., de Munno G., Sindona G. (2001). 9-Vinylguanine: an easy access to aza-analogs of 2′,3′-dideoxyguanosine. Tetrahedron.

[30] Hocek M., Holy A. (1995). A facile synthesis of 6-cyanopurine bases. Collect. Czech. Chem. Commun..

[31] Langli G., Gundersen L.-L., Rise F. (1996). Regiochemistry in Stille couplings of 2,6-dihalopurines. Tetrahedron.

[32] Brændvang M., Gundersen L.-L. (2007). Synthesis, biological activity and SAR of antimycobacterial 2- and 8-substituted 6-(2-furyl)-9-(*p*-methoxybenzyl)purines. Bioorg. Med. Chem..

[33] Toyota A., Katagiri N., Kaneko C. (1993). Mitsunobu reactions for the synthesis of carbocyclic analogues of nucleosides: Examination of the regioselectivity. Synth. Commun..

[34] Toyota A., Katagiri N., Kaneko C. (1993). Synthesis of nucleosides and related compounds. 31. The alkylation of 2-amino-6-chloropurine with alcohols by Mitsunobu reaction for a synthesis of carbocyclic guanosine analogs. Heterocycles.

[35] Maruyama T., Kozai S., Uchida M. (1999). Synthesis of *N*-aryl uracils and hypoxanthines and their biological properties. Nucleosides Nucleotides.

[36] Lu W., Sengupta S., Peterson J.L., Akhmedow N.G., Shi X. (2007). Mitsunobu coupling of nucleobases and alcohols: An efficient, practical synthesis for novel nonsugar carbon nucleosides. J. Org. Chem..

[37] Trost B.M., Madsen R., Guile S.G., Brown B. (2000). Palladium-catalyzed enantioselective synthesis of carbanucleosides. J. Am. Chem. Soc..

[38] Choo H., Chong Y., Chu C.K. (2001). Solid phase synthesis of carbocyclic l-2′-deoxynucleosides. Org. Lett..

[39] Guillarme S., Legoupy S., Aubertin A.-M., Olicard C., Bourgougnon N., Huet F. (2003). Rapid access to acyclic nucleosides via conjugate addition. Tetrahedron.

[40] Velcicky J., Lanver A., Lex J., Prokop A., Wieder T., Schmalz H.-G. (2004). Transition-metal-mediated synthesis of novel carbocyclic nucleoside analogues with antitumoral activity. Chem. Eur. J..

[41] Pautus S., Sehr P., Lewis J., Fortune A., Wolkerstorfer A., Szolar O., Guilligay D., Lunardi T., Decout J.-L., Cusack S. (2013). New 7-methylguanine derivatives targeting the influenza polymerase PB2 Cap-binding domain. J. Med. Chem..

[42] Roberts J.D., Chambers V.C. (1951). Small-ring compounds. VIII. Some nucleophilic displacement reactions of cyclopropyl, cyclobutyl, cyclopentyl and cyclohexyl *p*-toluenesulfonates and halides. J. Am. Chem. Soc..

[43] Gundersen L.-L. (2008). Metal-mediated C–C and C–N bond formation in the synthesis of bioactive purines. Targets Heterocycl. Syst..

[44] Kobayashi S., Kawamoto T., Uehara S., Fukuyama T., Ryu I. (2010). Black-light-induced radical/ionic hydroxymethylation of alkyl iodides with atmospheric CO in the presence of tetrabutylammonium borohydride. Org. Lett..

[45] Gamadeku T., Gundersen L.-L. (2010). Synthesis of 8-bromo-*N*-benzylpurines via 8-lithiated purines; Scopes and Limitations. Synth. Commun..

[46] Marzouk V.H.R., Hennum M., Gundersen L.-L. (2013). Efficient synthesis of cytotoxic pyrido[1,2-*e*]purines from purines employing direct C-allylation and RCM-oxidation as key-steps. Tetrahedron Lett..

[47] Ikehara M., Tada H., Muneyama K. (1965). Synthesis of 8-hydroxypurine nucleosides. Chem. Pharm. Bull..

[48] Jang M.-Y., Lin Y., de Jonghe S., Gao L.-J., Vanderhoydonck B., Froeyen M., Rozenski J., Herman J., Louat T., van Belle K. (2011). Discovery of 7-*N*-piperazinylthiazolo[5,4-*d*]pyrimidine analogues as a novel class of immunosuppressive agents with *in vivo* biological activity. J. Med. Chem..

[49] Hagiya K., Muramoto N., Misaki T., Sugimura T. (2009). DMEAD: A new dialkyl azodicarboxylate for the Mitsunobu reaction. Tetrahedron.

[50] Fromme J.C., Bruner S.D., Yang W., Karplus M., Verdine G.L. (2003). Product-assisted catalysis in base-excision DNA repair. Nat. Struct. Biol..

[51] Winstein S., Grunwald E., Buckles R.E., Hanso C. (1948). The role of neighboring groups in replacement reactions. XI. Some reactivities involving neighboring groups. J. Am. Chem. Soc..

[52] Forkel N.V., Henderson D.A., Fuchter M.J. (2012). Lanthanide replacement in organic synthesis: Luche-type reduction of α,β-unsaturated ketones in the presence of calcium triflate. Green Chem..

[53] Jacquet O., Bergholz T., Magnier-Bouvier C., Mellah M., Guillot R., Fiaud J.-C. (2010). Palladium-catalyzed and samarium-promoted coupling of stereochemically-biased allylic acetates with carbonyl compounds. Tetrahedron.

[54] Kim K., McComas W. (2000). Chemoselective high-throughput purification mediated by solid-supported reagents: Its application to the first 6,9-disubstituted purine library synthesis. Comb. Chem. High Throughput Screen..

[55] Legraverend M., Ludwig O., Bisagni E., Leclerc S., Meijer L., Giocanti N., Sadri R., Favaudon V. (1999). Synthesis and *in vitro* evaluation of novel 2,6,9-trisubstituted purines acting as cyclin-dependent kinase inhibitors. Bioorg. Med. Chem..

[56] Lukin K.A., Yang C.X., Bellettini J.R., Narayanan B.A. (2000). New purine derivatives as efficient preparation of nucleoside analogs via alkylation. Nucleosides Nucleotides Nucleic Acids.

[57] Gültekin Z. (2006). Palladium-catalyzed synthesis of 9-(2-cyclopentenyl)guanine. Asian J. Chem..

