# Supplementary material for: Synthetic Routes to N-9 Alkylated 8-Oxoguanines; Weak Inhibitors of the Human DNA Glycosylase OGG1
Source: Molecules. 2015 Sep 2;20(9):15944–65. doi: 10.3390/molecules200915944 (PMC6332111; doi:10.3390/molecules200915944)
Supplement: Supplementary file 1 [file molecules-20-15944-s001.pdf]

Supplementary Materials

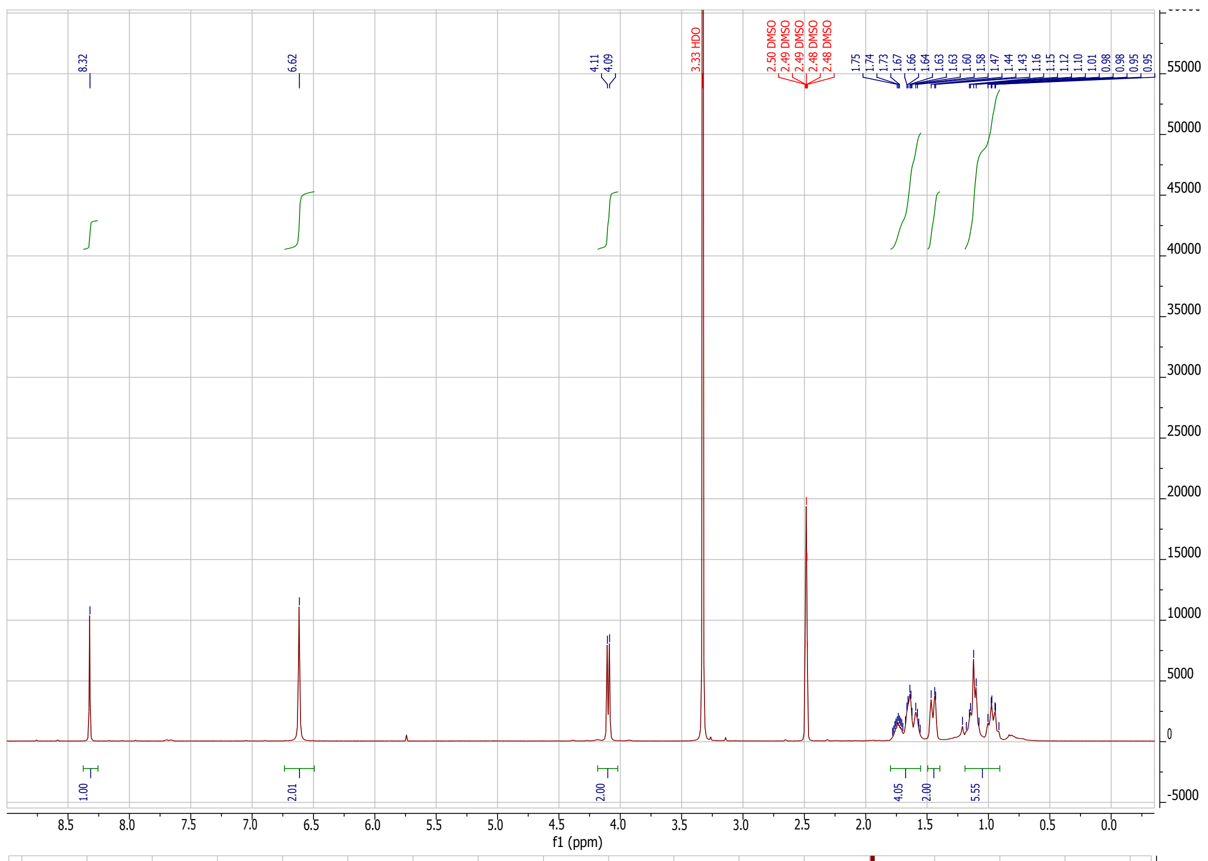

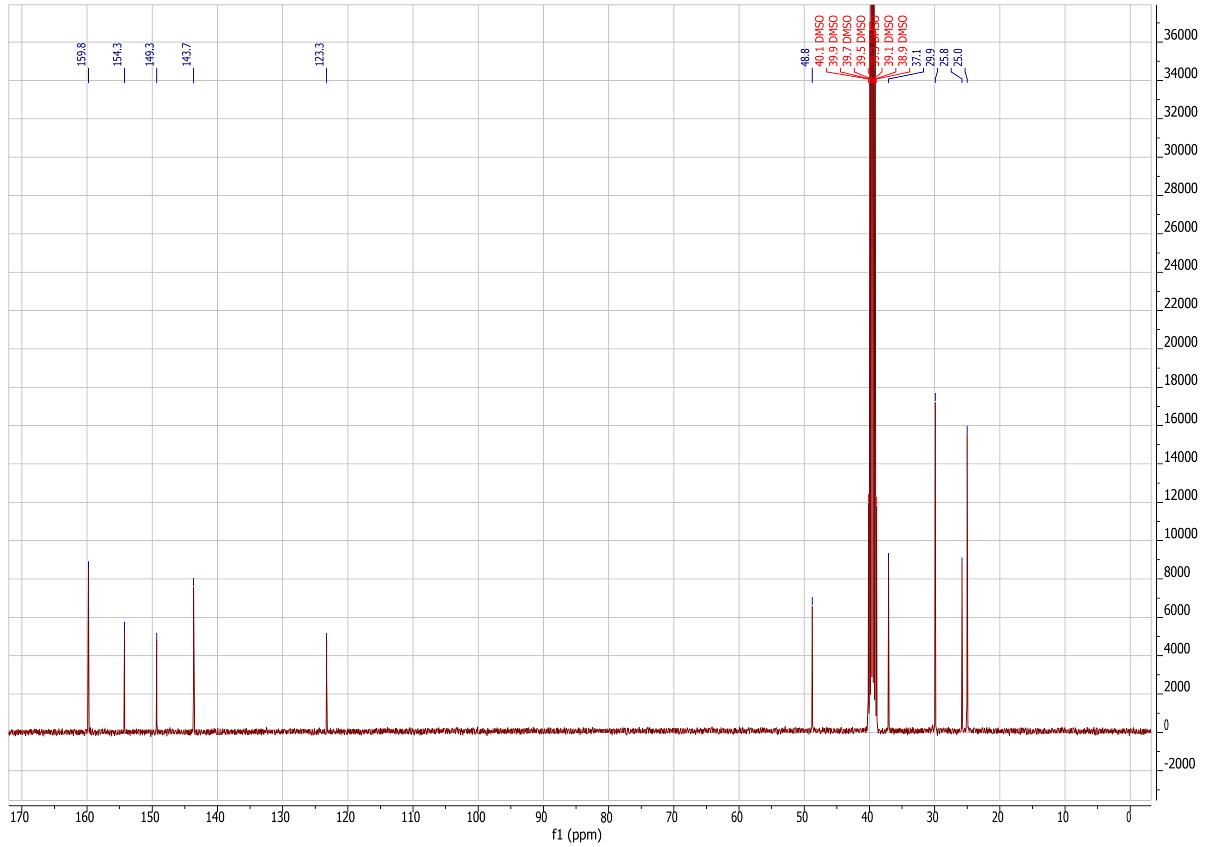

Figure S1. ^{1}H-NMR and ^{13}C-NMR of 2-Amino-6-chloro-7-(cyclohexylmethyl)-7*H*-purine (**3a**).

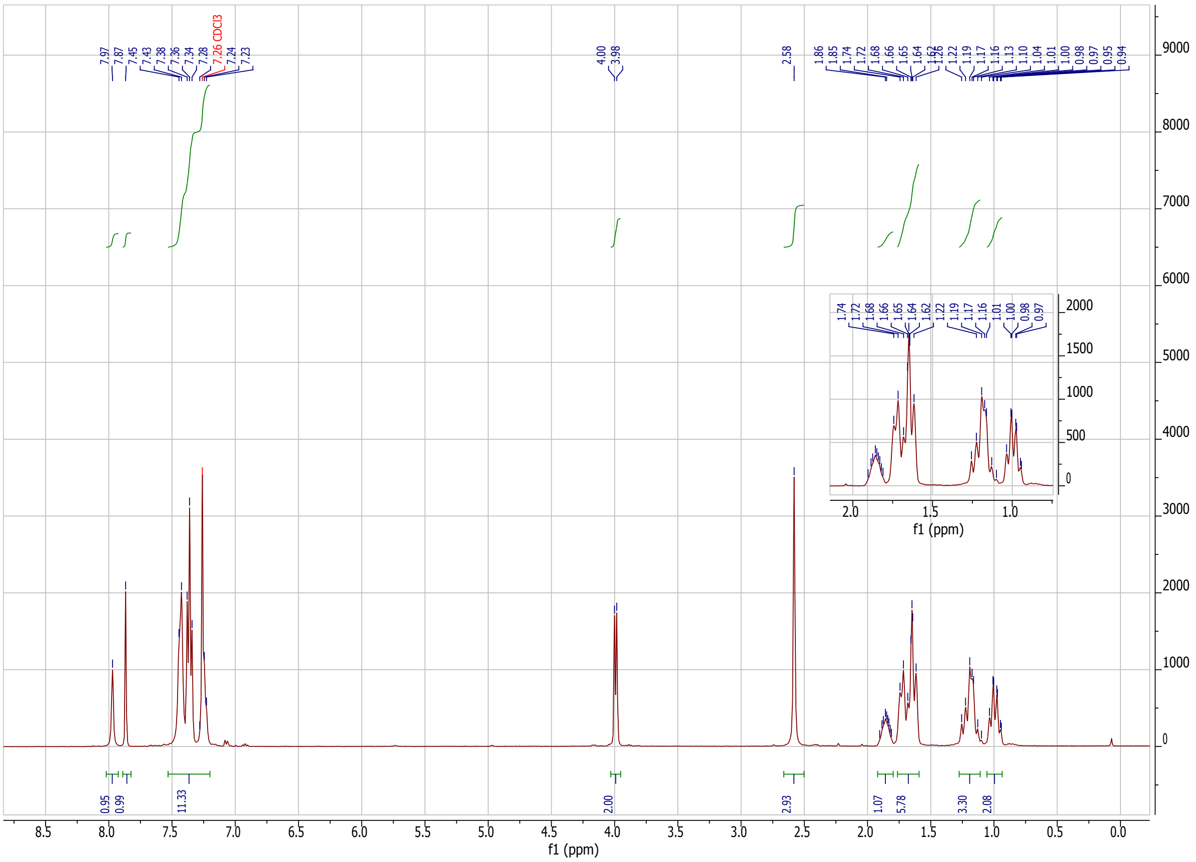

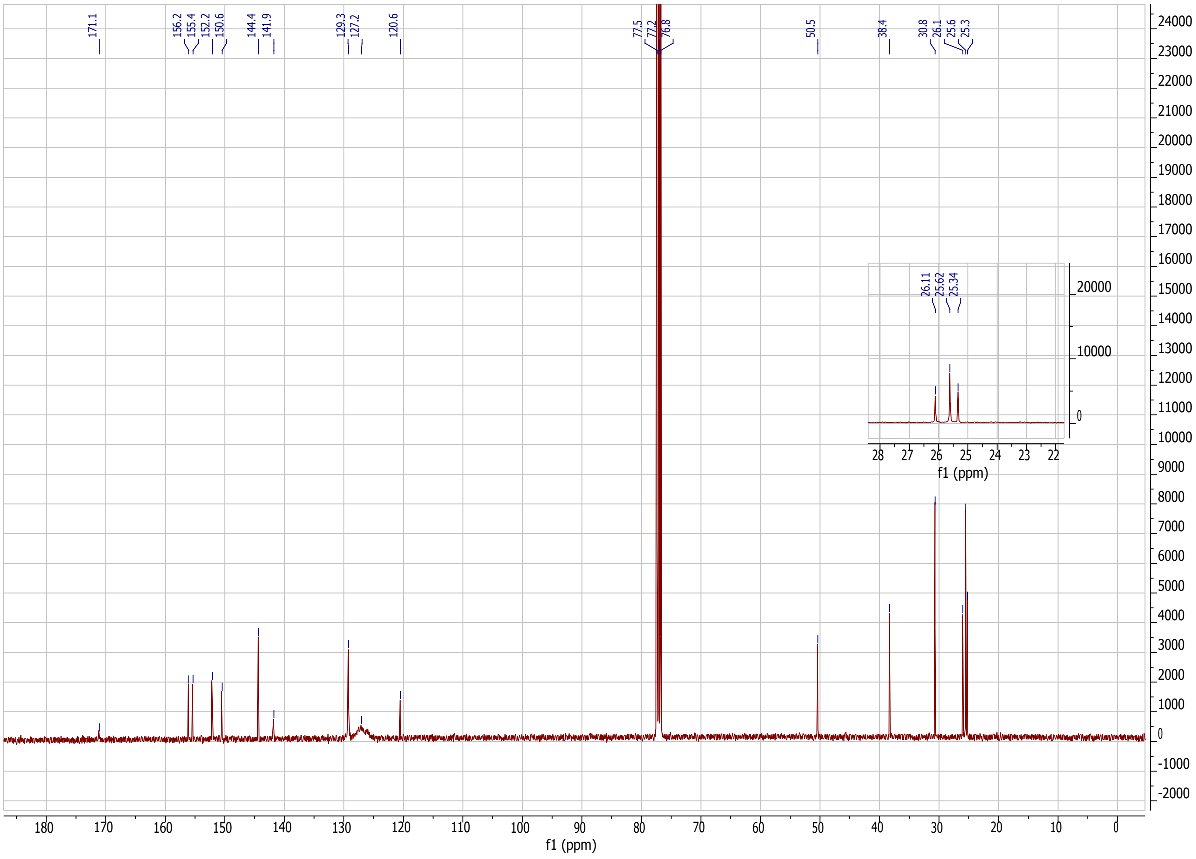

Figure S2. ^{1}H-NMR and ^{13}C-NMR 2-Acetamido-9-(cyclohexylmethyl)-9*H*-purin-6-yl diphenylcarbamate (**2e**).

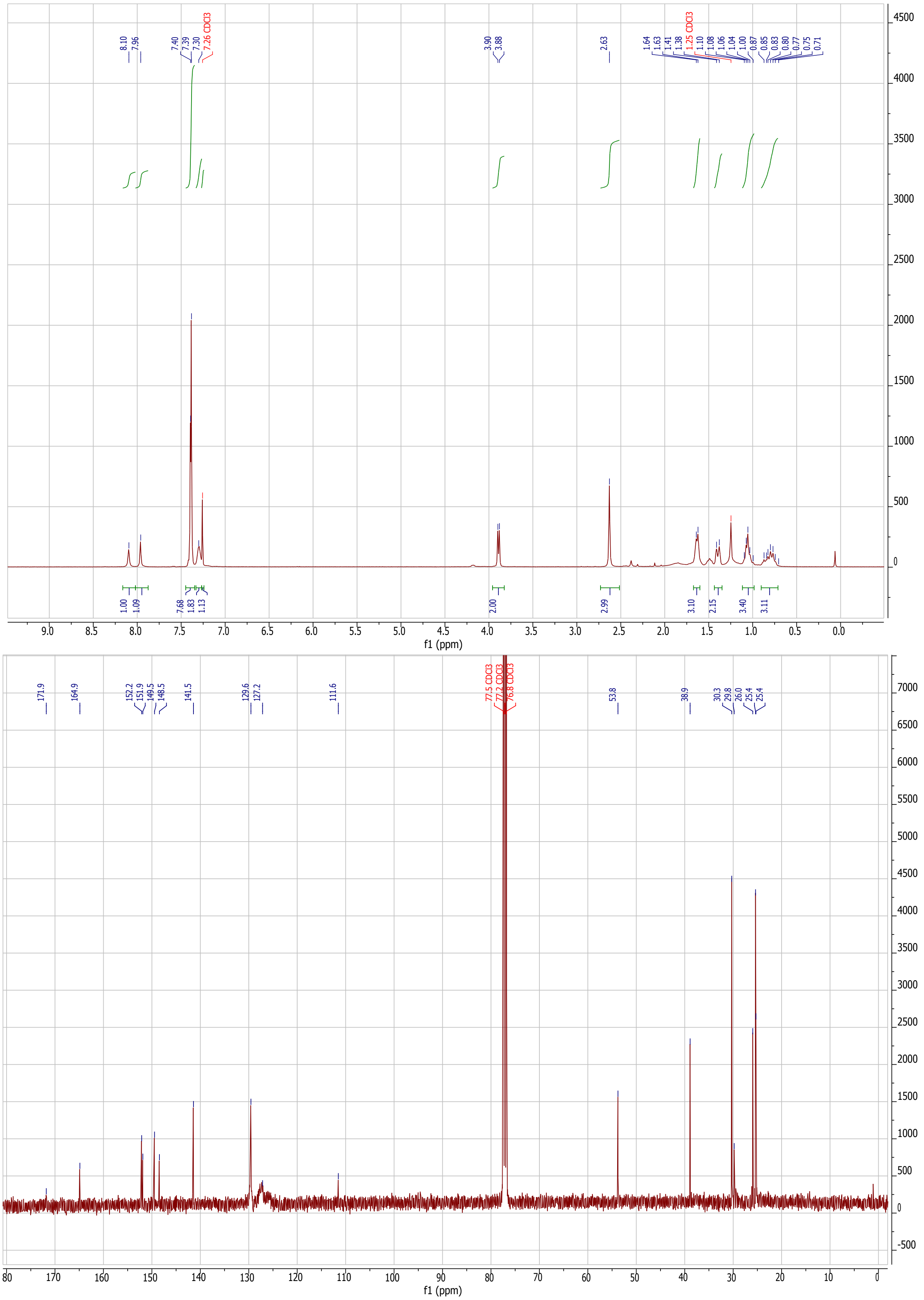

Figure S3. ^{1}H-NMR and ^{13}C-NMR 2-Acetamido-7-(cyclohexylmethyl)-7*H*-purin-6-yl diphenylcarbamate (**3e**).

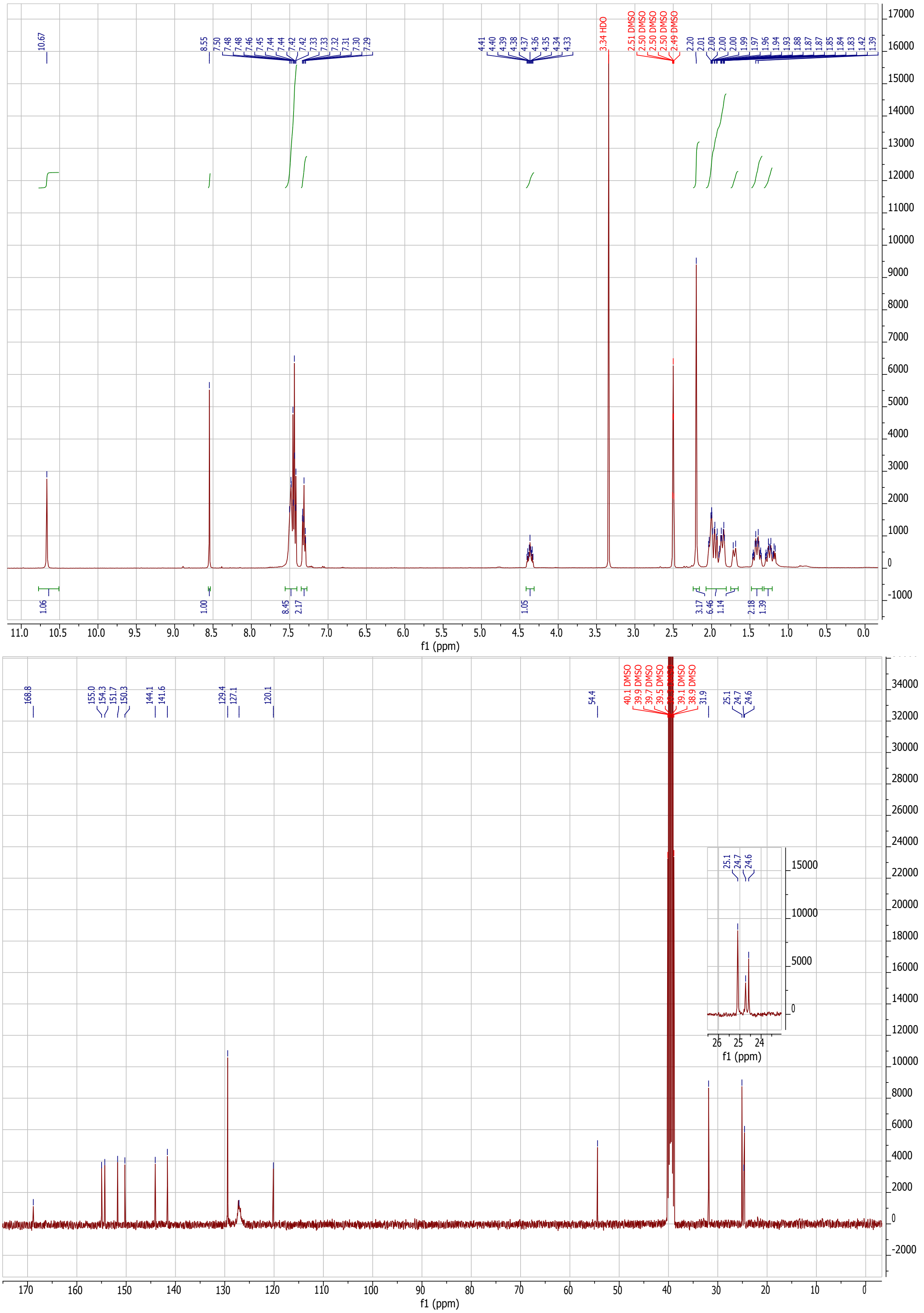

Figure S4. ^{1}H-NMR and ^{13}C-NMR 2-Acetamido-9-(cyclohexyl)-9*H*-purin-6-yl diphenylcarbamate (**2f**).

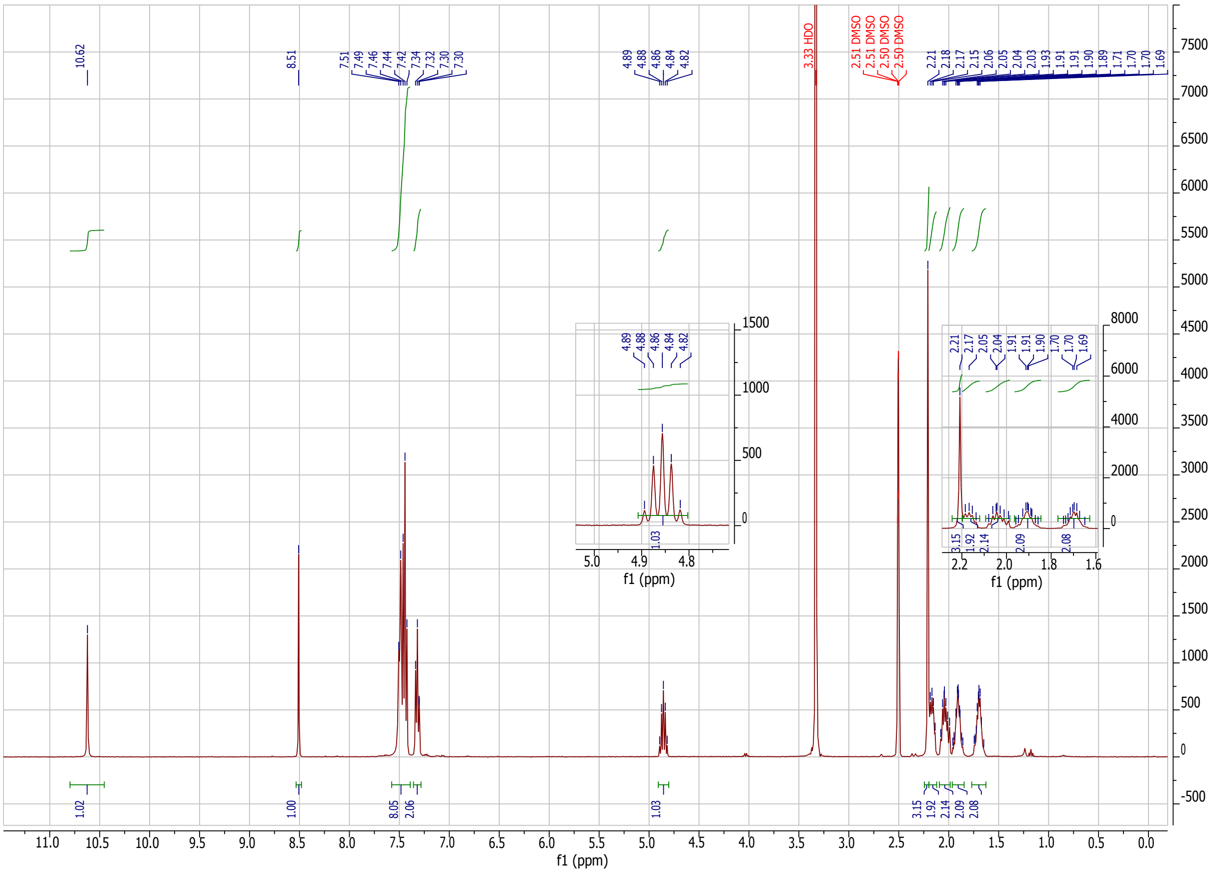

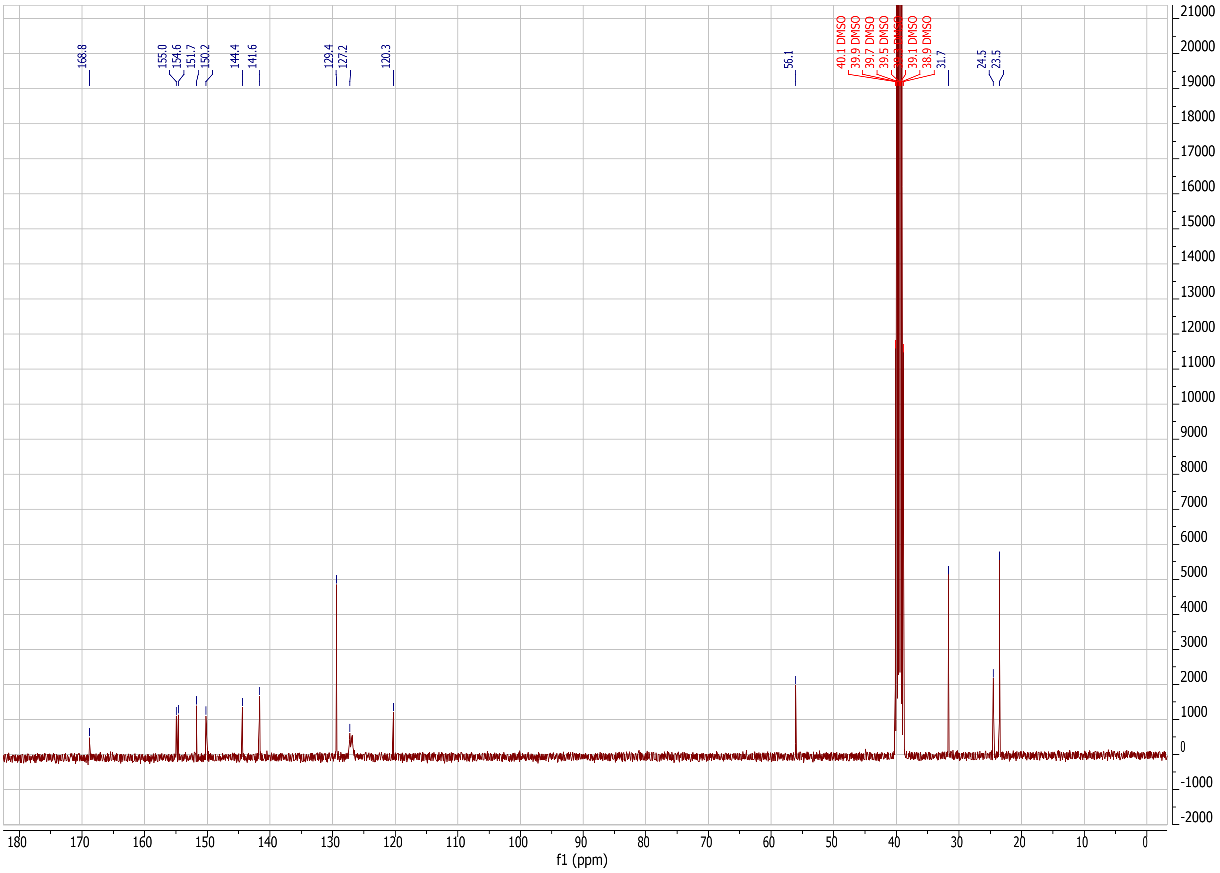

Figure S5. ^{1}H-NMR and ^{13}C-NMR 2-Acetamido-9-(cyclopentyl)-9*H*-purin-6-yl diphenylcarbamate (**2g**).

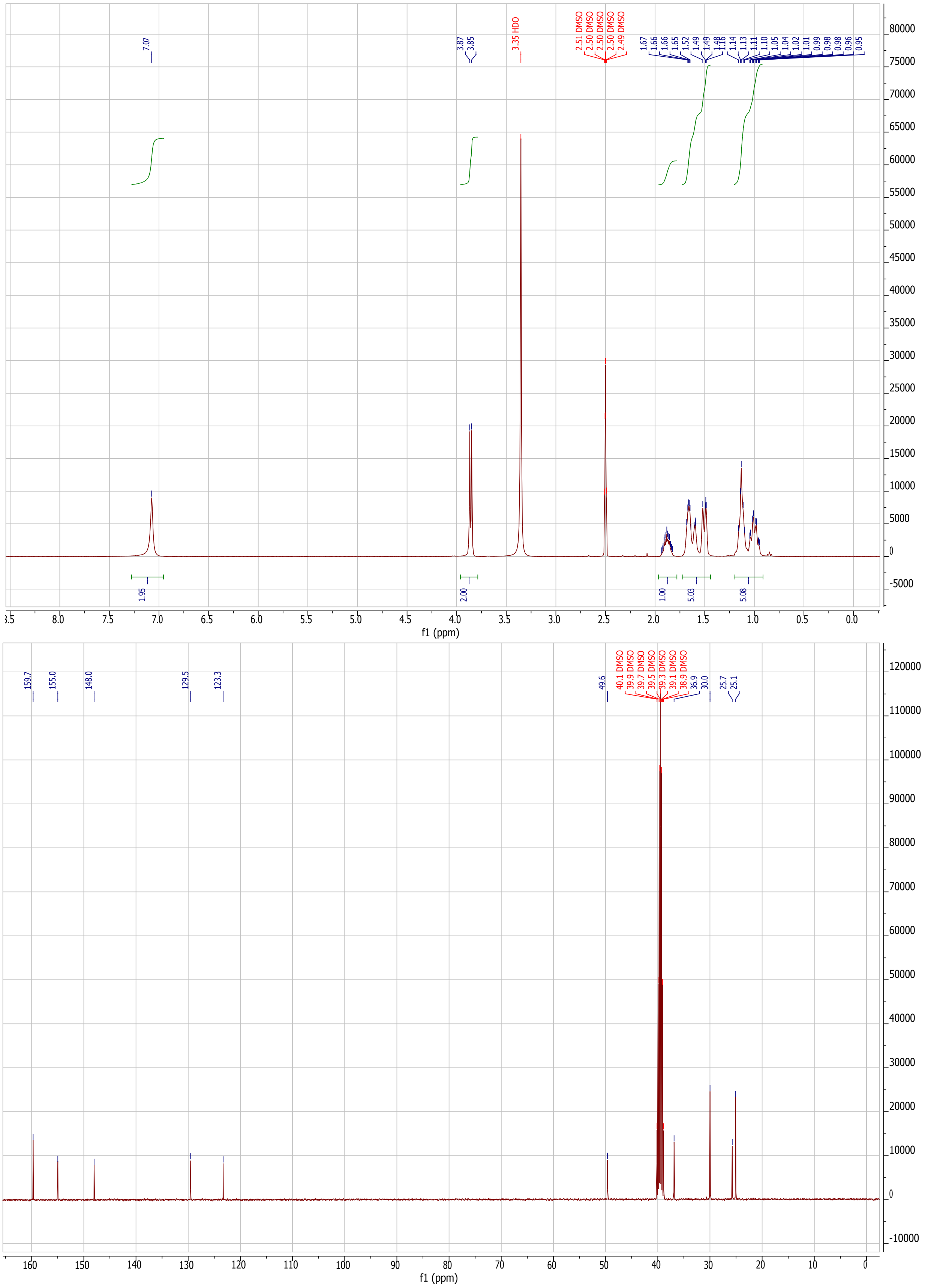

Figure S6. ^{1}H-NMR and ^{13}C-NMR 2-Amino-8-bromo-6-chloro-9-(cyclohexylmethyl)-9*H*-purine (**4a**).

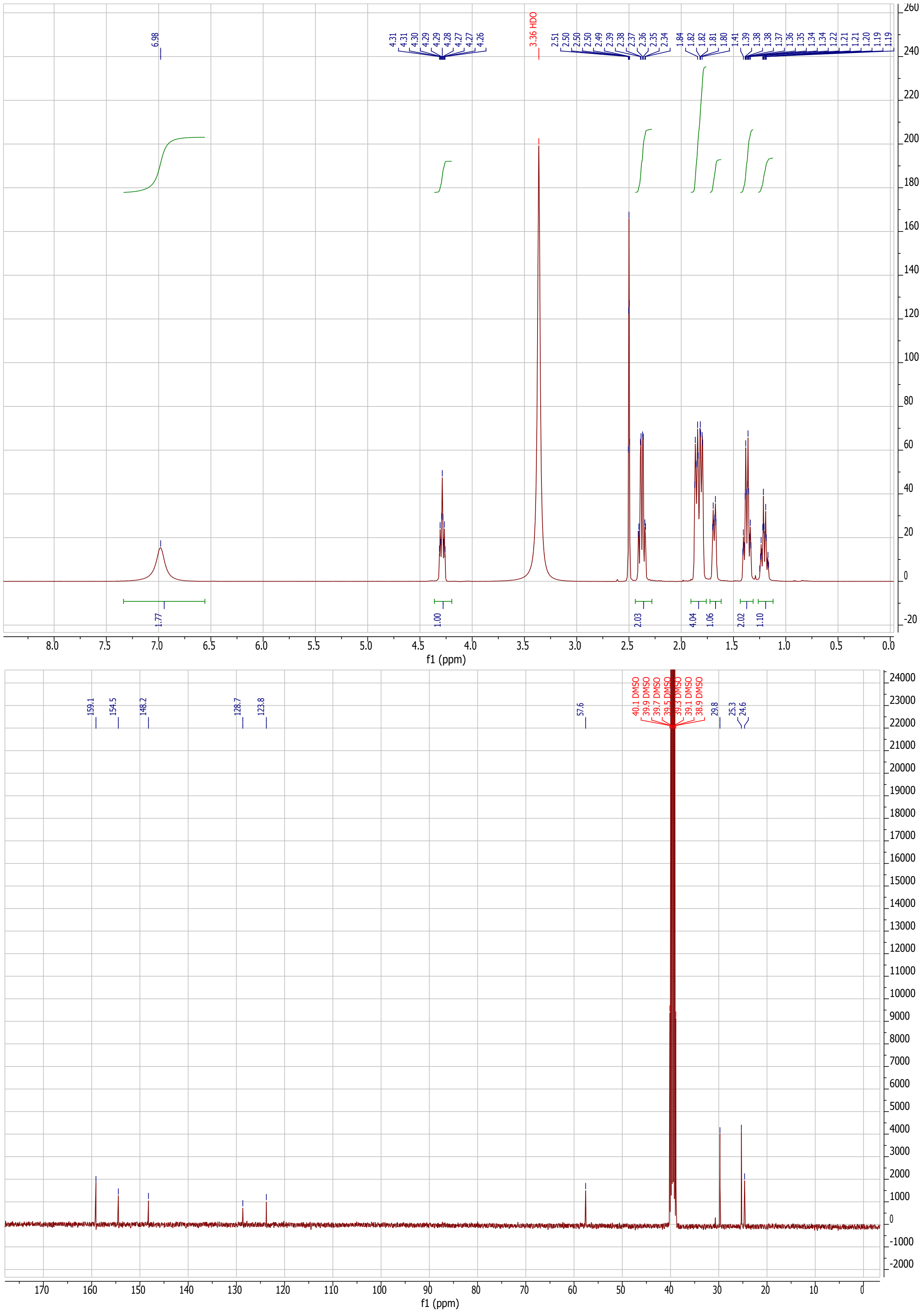

Figure S7. ^{1}H-NMR and ^{13}C-NMR 2-Amino-8-bromo-6-chloro-9-(cyclohexyl)-9*H*-purine (**4b**).

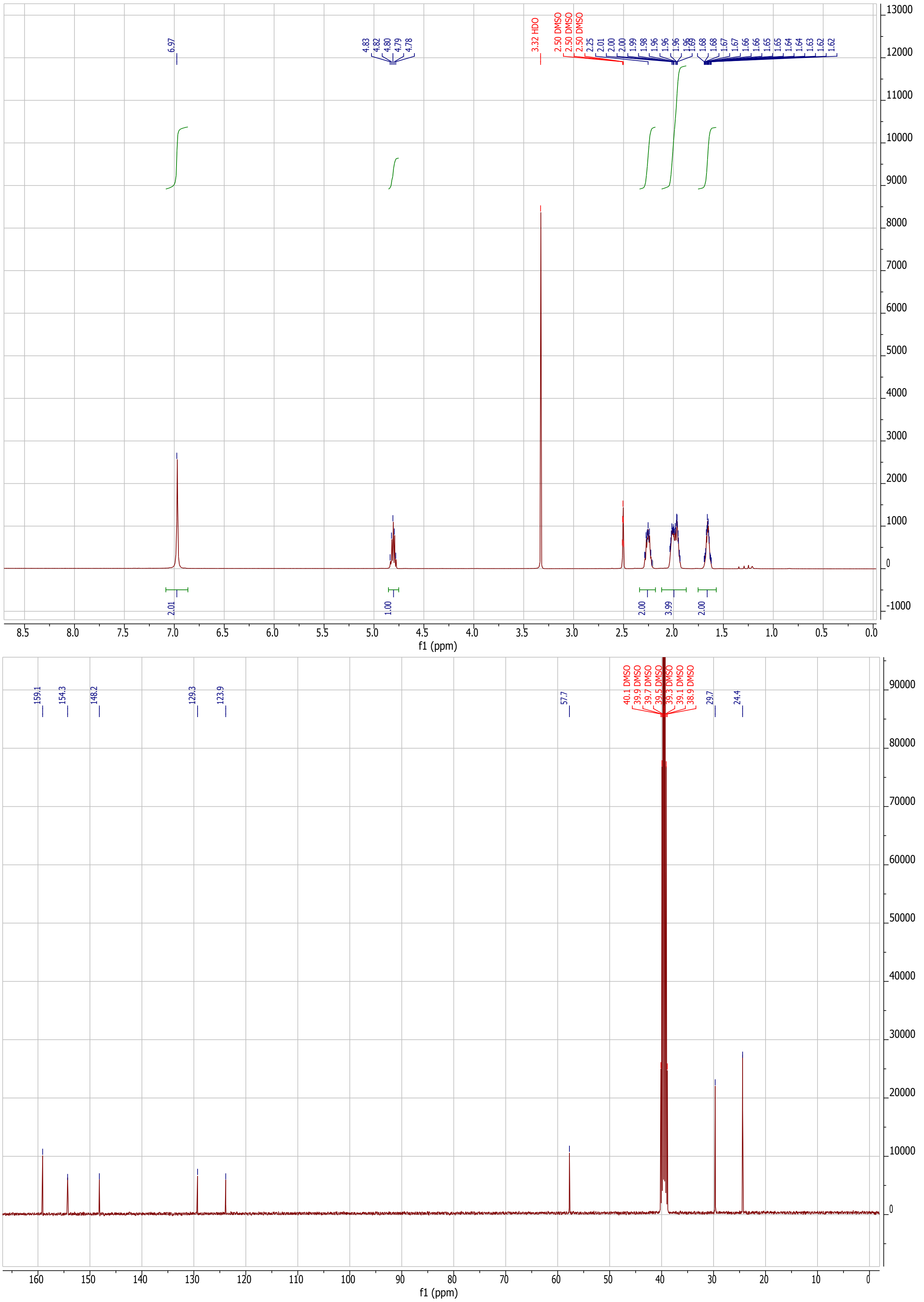

Figure S8. ^{1}H-NMR and ^{13}C-NMR 2-Amino-8-bromo-6-chloro-9-(cyclopentyl)-9*H*-purine (**4c**).

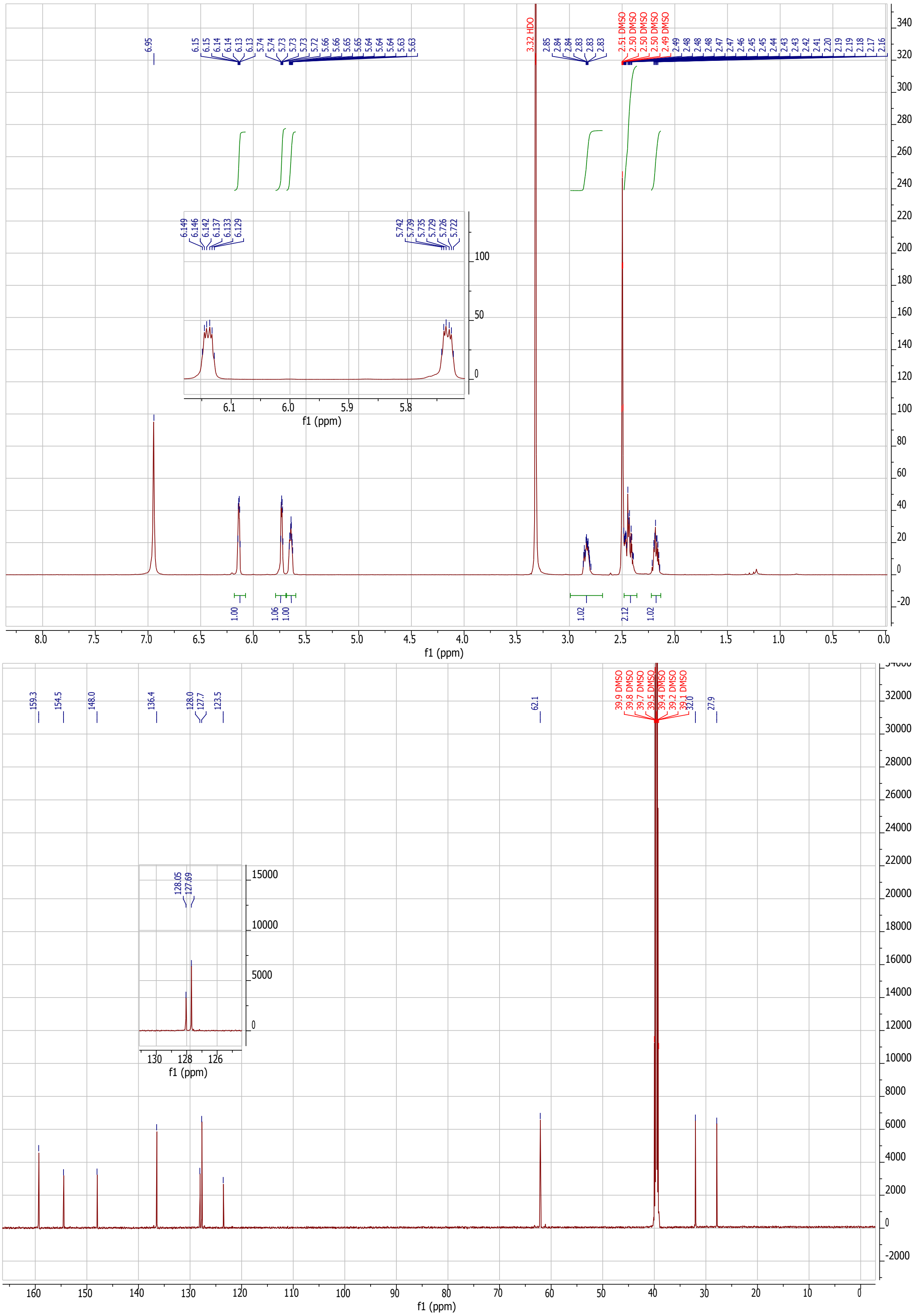

Figure S9. ^{1}H-NMR and ^{13}C-NMR 2-Amino-8-bromo-6-chloro-9-(cyclopent-2-enyl)-9*H*-purine (**4d**).

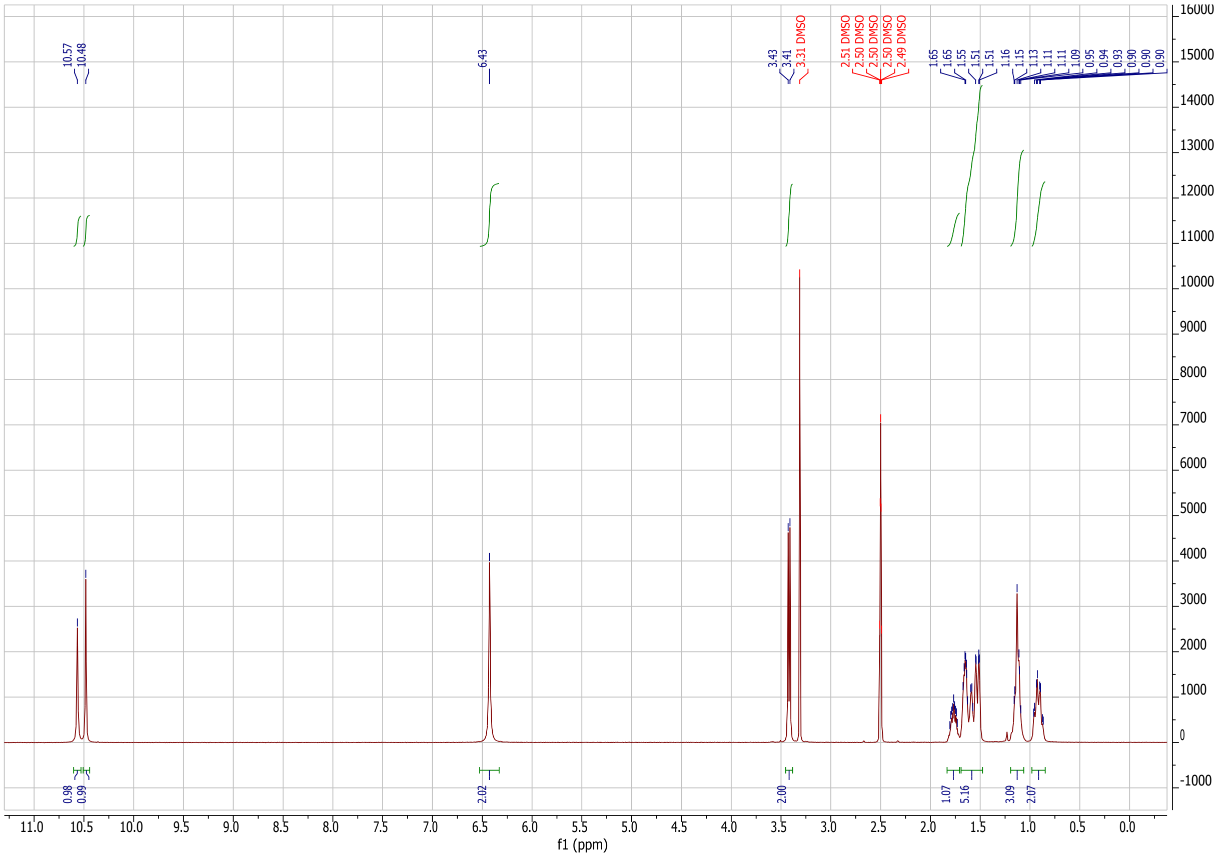

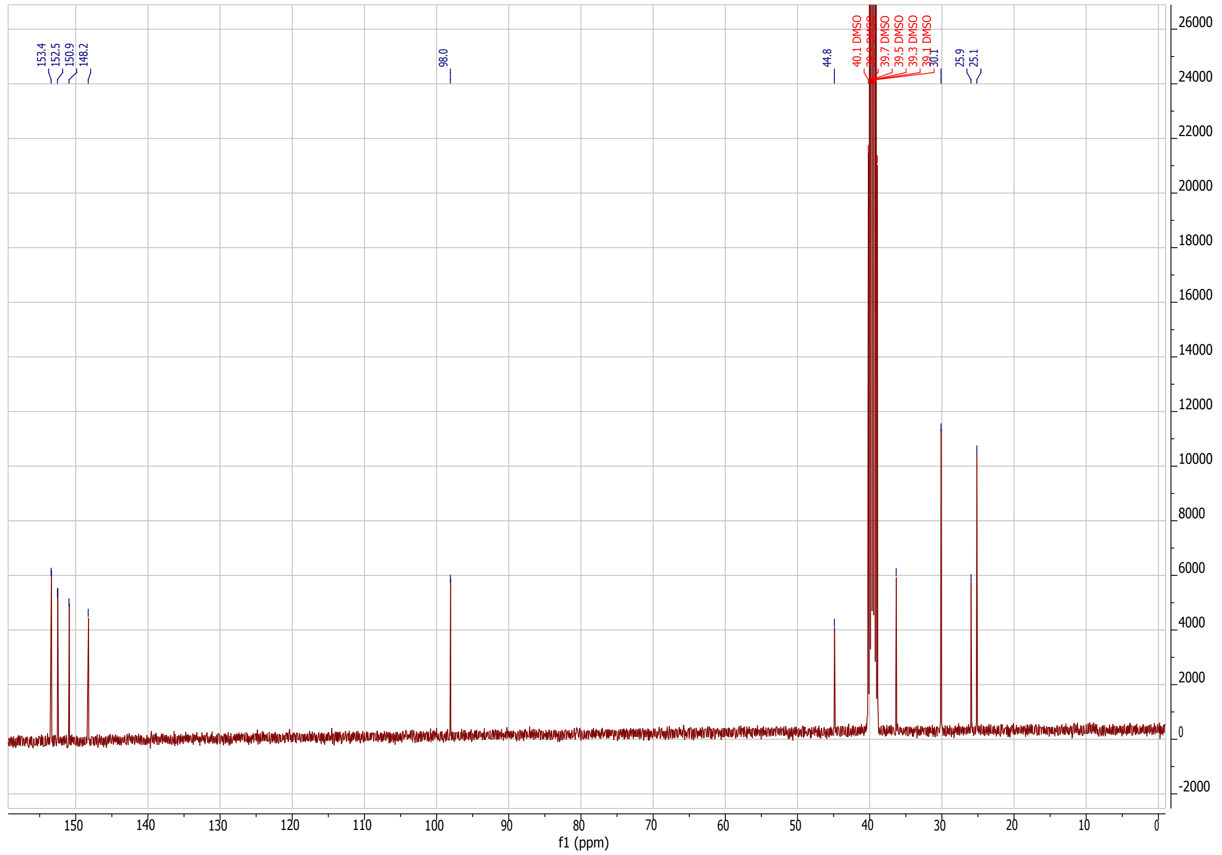

Figure S10. ^{1}H-NMR and ^{13}C-NMR 9-(Cyclohexylmethyl)-8-oxoguanine (**5a**).

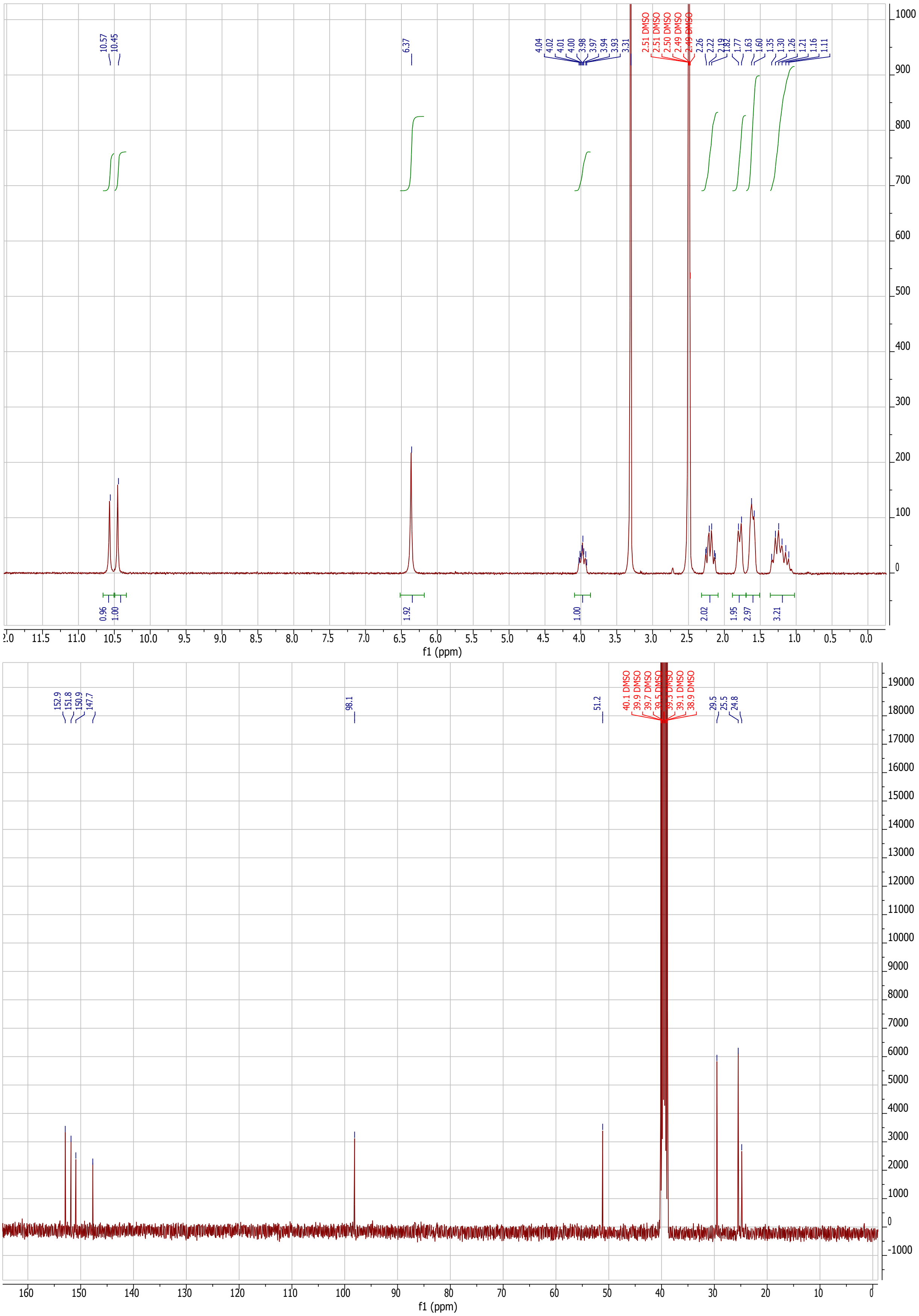

Figure S11. ^{1}H-NMR and ^{13}C-NMR 9-(Cyclohexyl)-8-oxoguanine (**5b**).

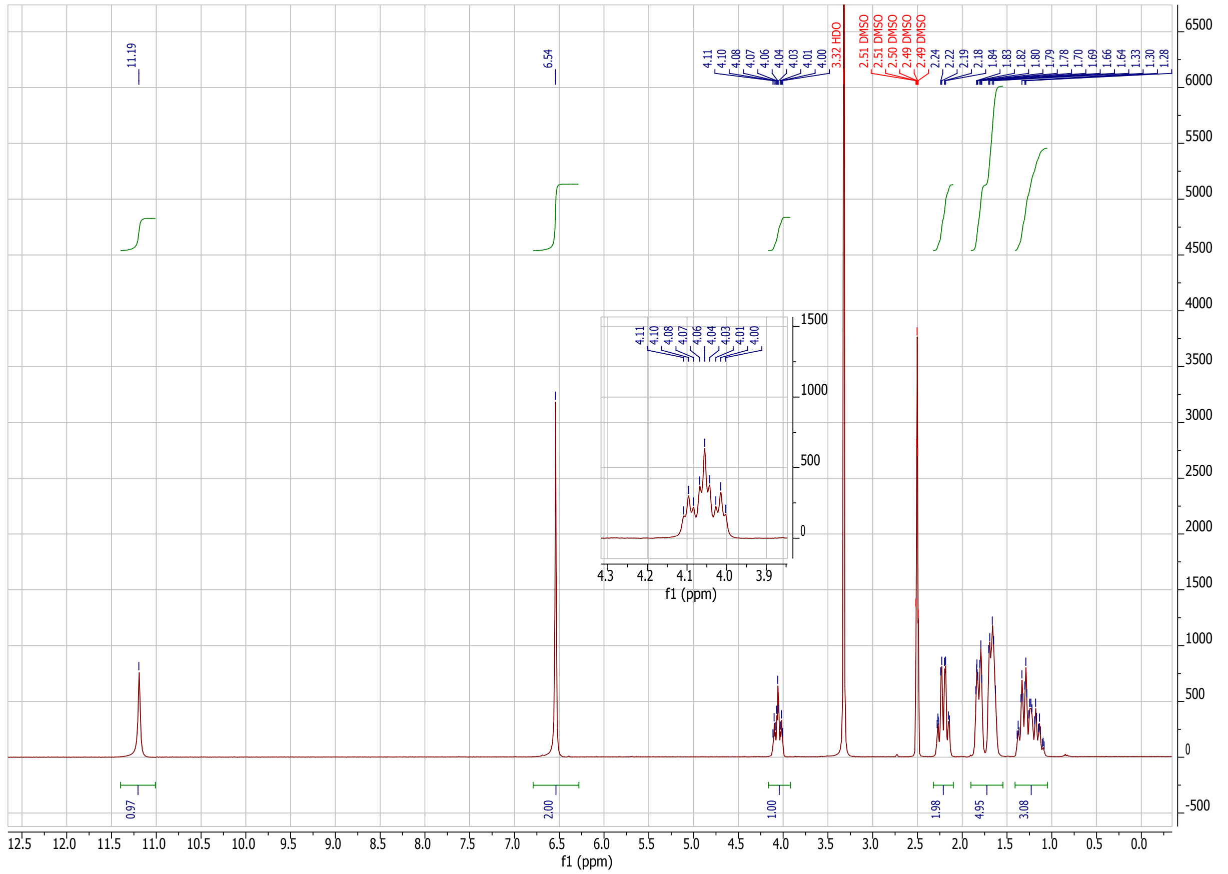

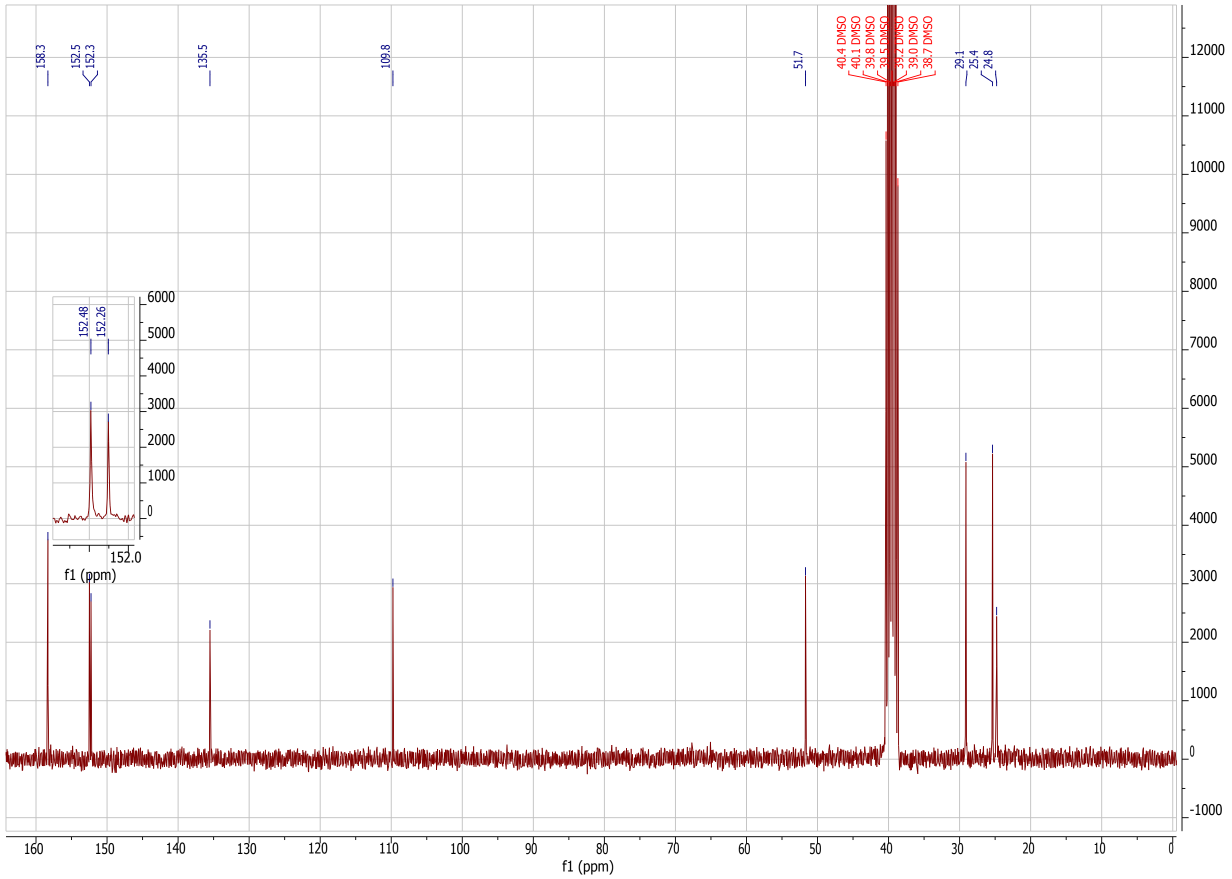

Figure S12. ^{1}H-NMR and ^{13}C-NMR 2-Amino-6-chloro-9-cyclohexyl-7*H*-purin-8(9*H*)-one (**6b**).

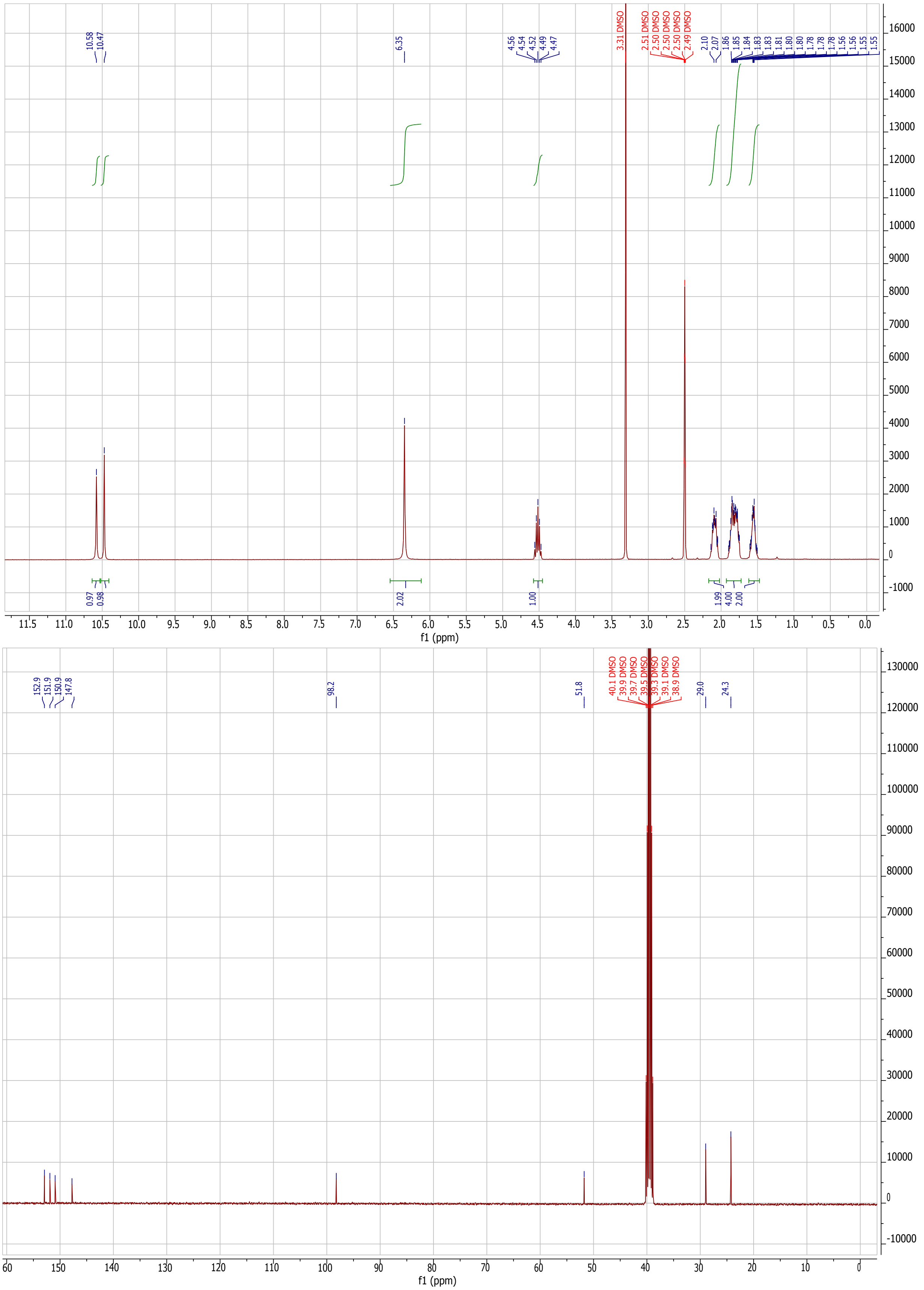

Figure S13. ^{1}H-NMR and ^{13}C-NMR 9-(Cyclopentyl)-8-oxoguanine (**5c**).

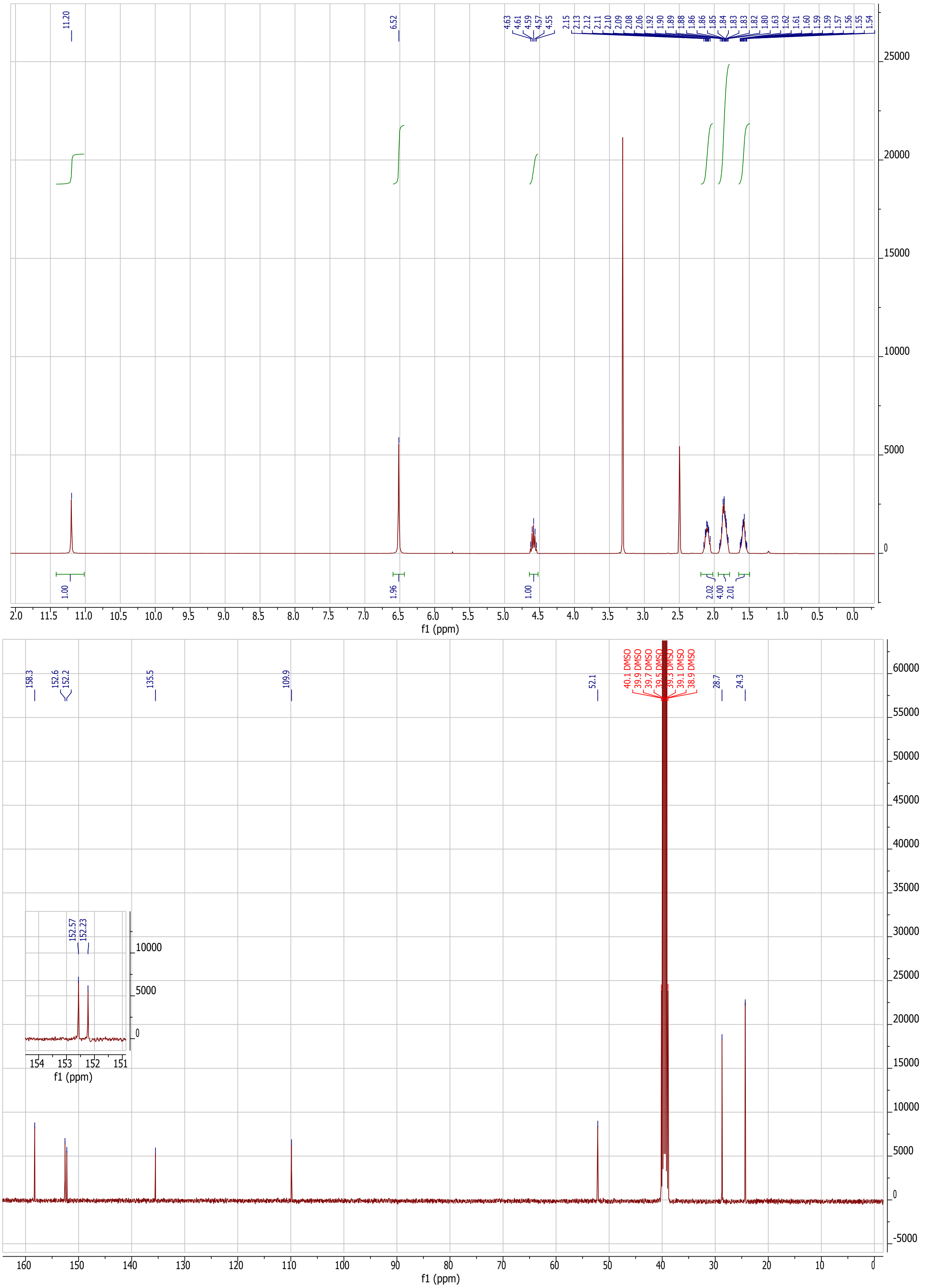

Figure S14. ^{1}H-NMR and ^{13}C-NMR 2-Amino-6-chloro-9-cyclopentyl-7*H*-purin-8(9*H*)-one (**6c**).

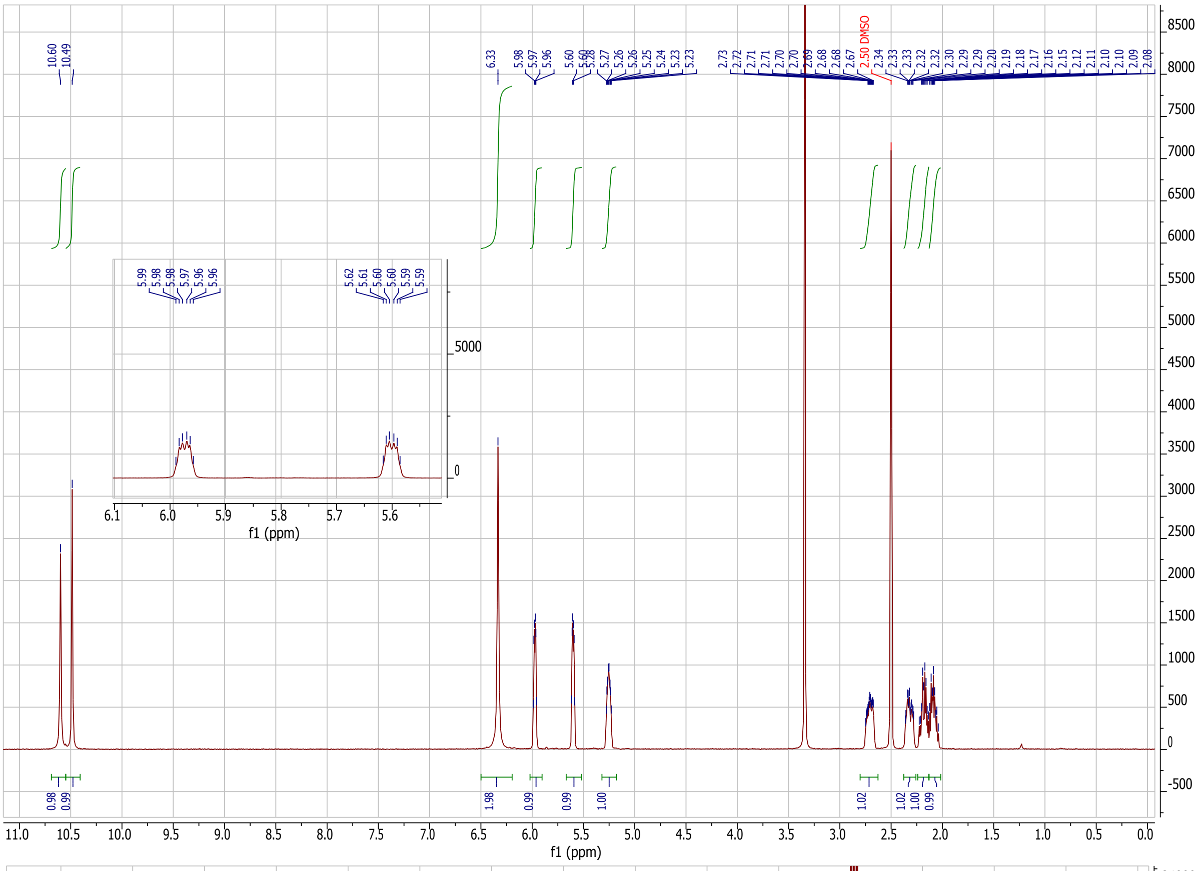

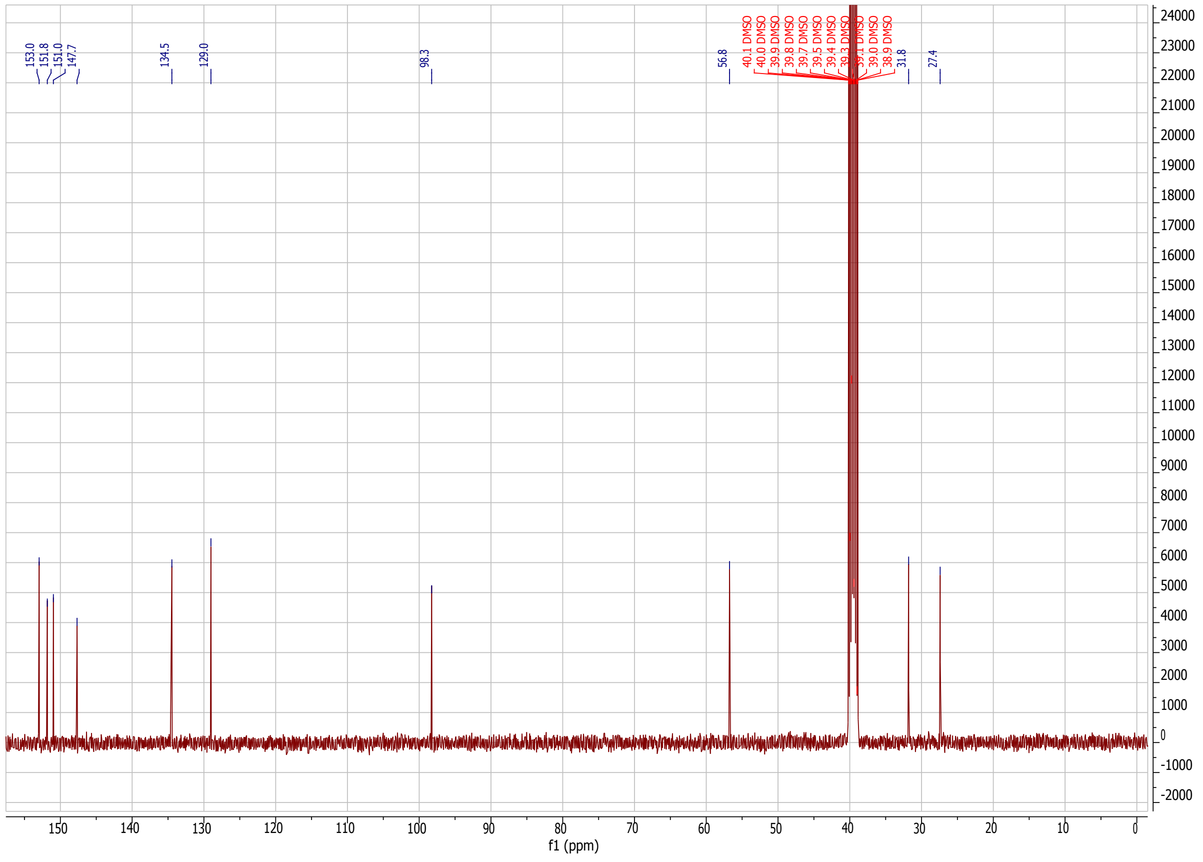

Figure S15. ^{1}H-NMR and ^{13}C-NMR 9-(Cyclopent-2-enyl)-8-oxoguanine (**5d**).

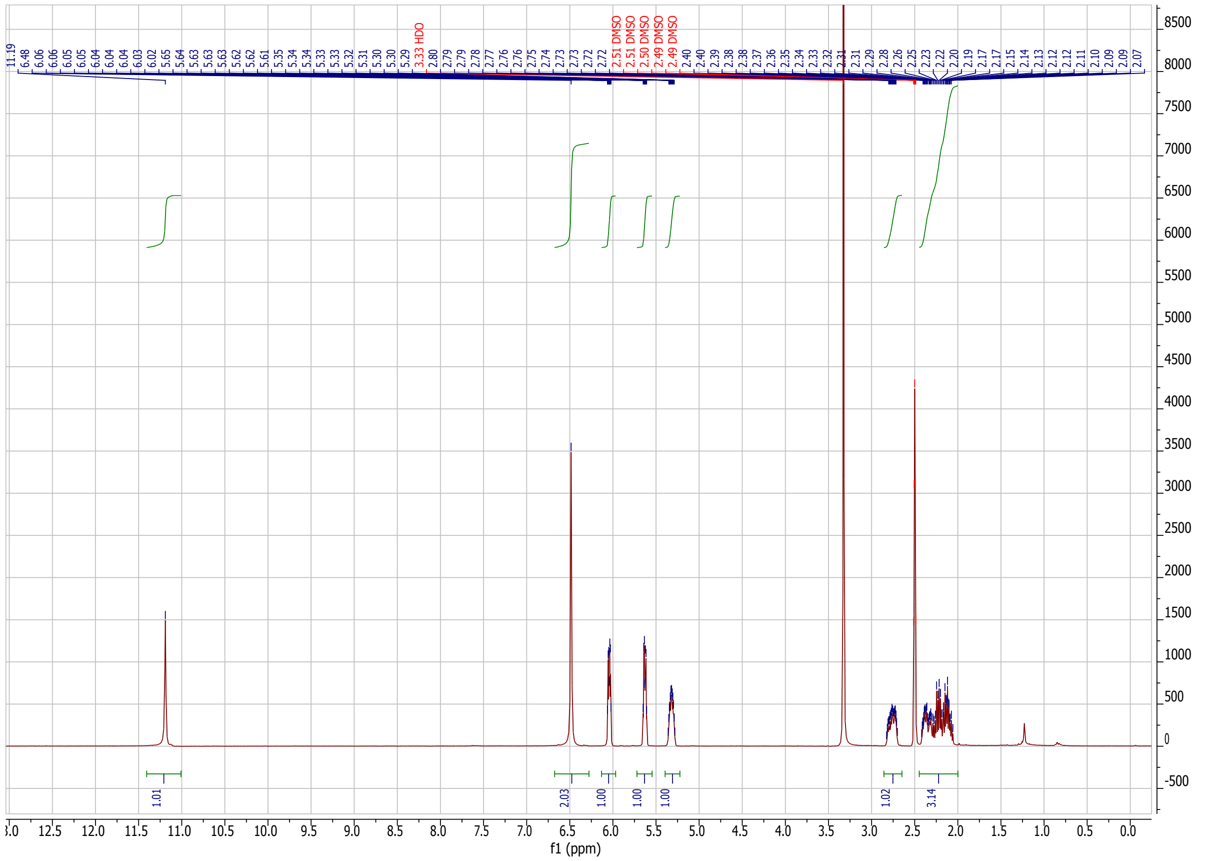

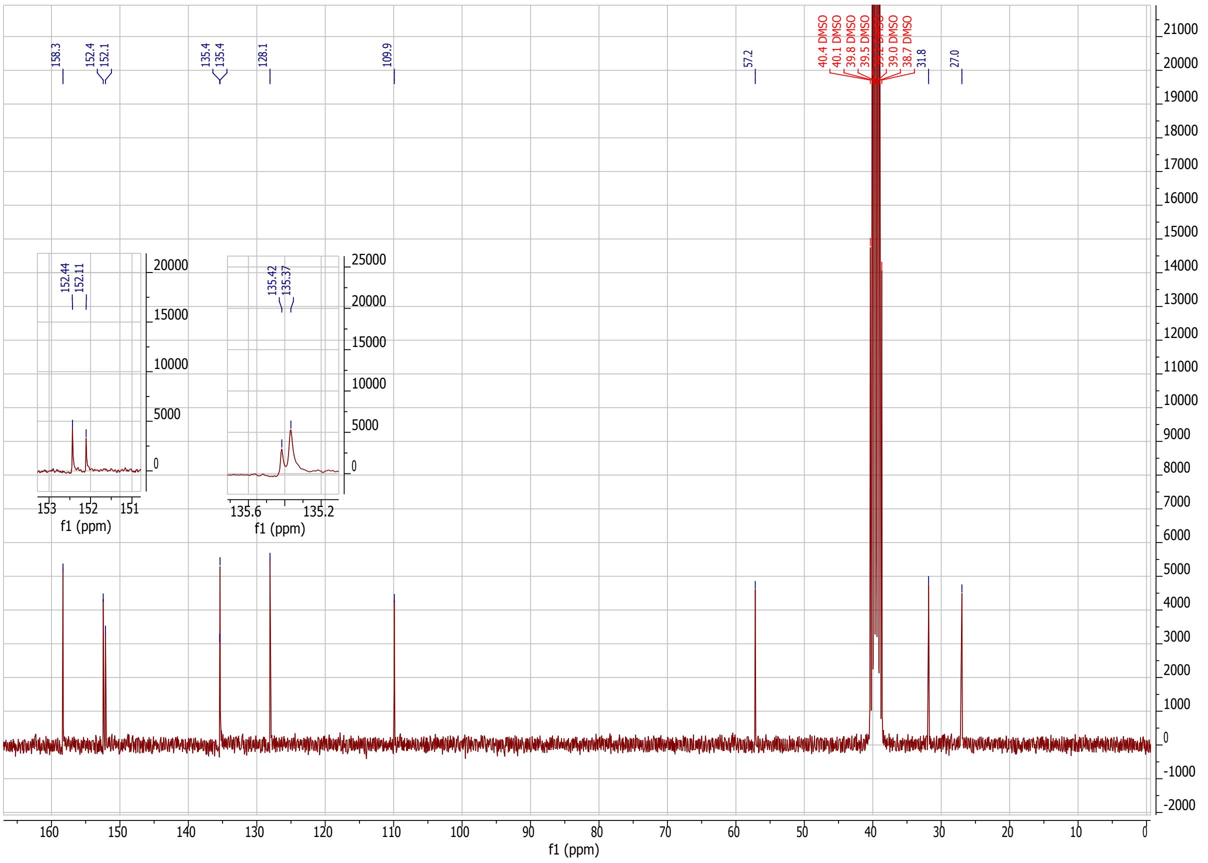

Figure S16. ^{1}H-NMR and ^{13}C-NMR 2-Amino-6-chloro-9-(cyclopent-2-enyl)-7*H*-purin-8(9*H*)-one (**6d**).

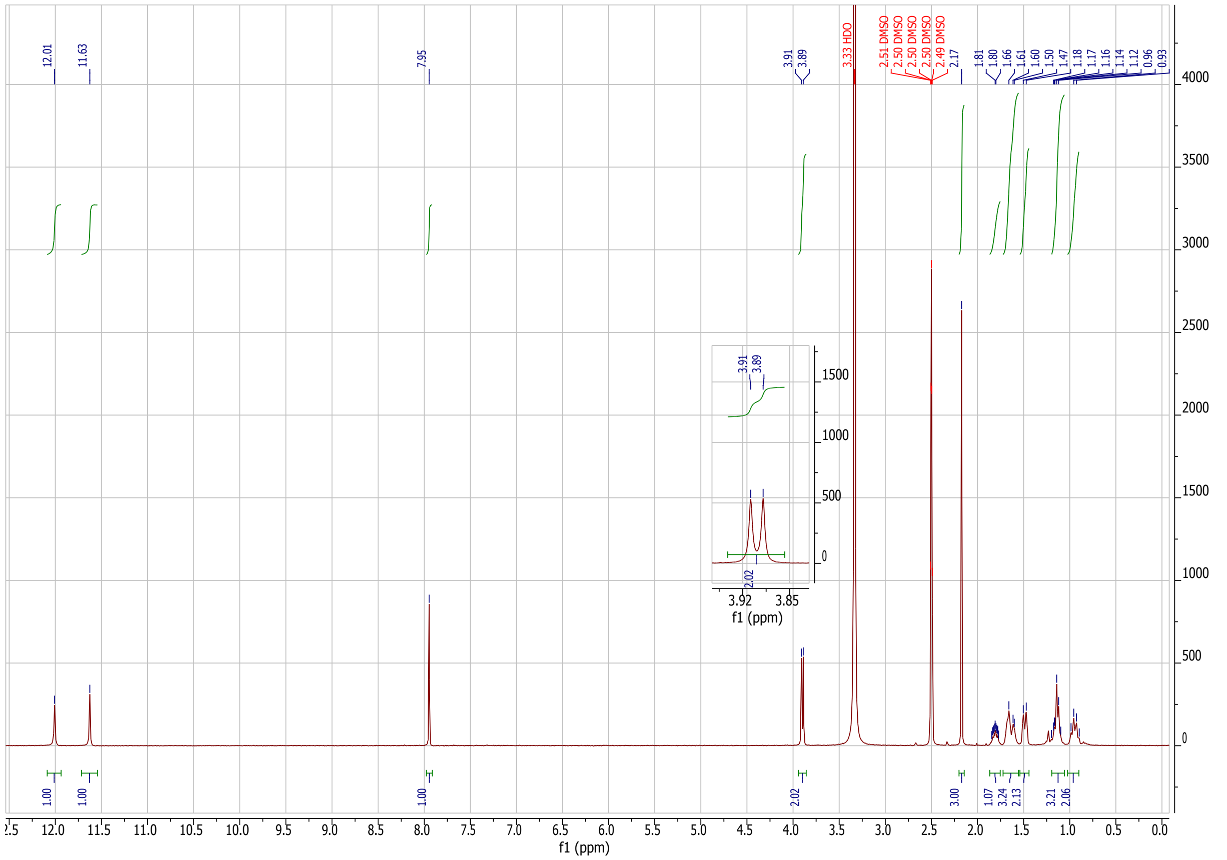

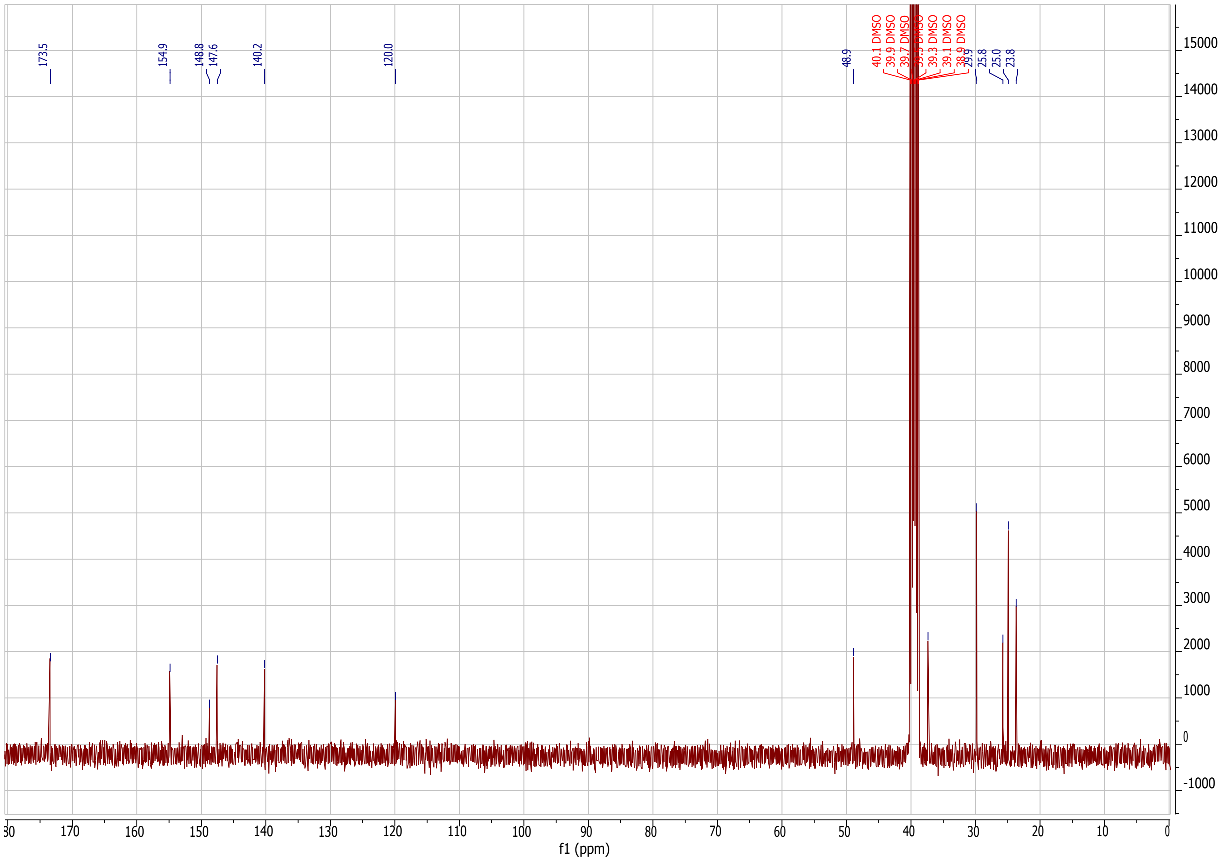

Figure S17. ^{1}H-NMR and ^{13}C-NMR *N*-[9-(Cyclohexylmethyl)-6-oxo-6,9-dihydro-1*H*-purin-2-yl]acetamide (**7**).

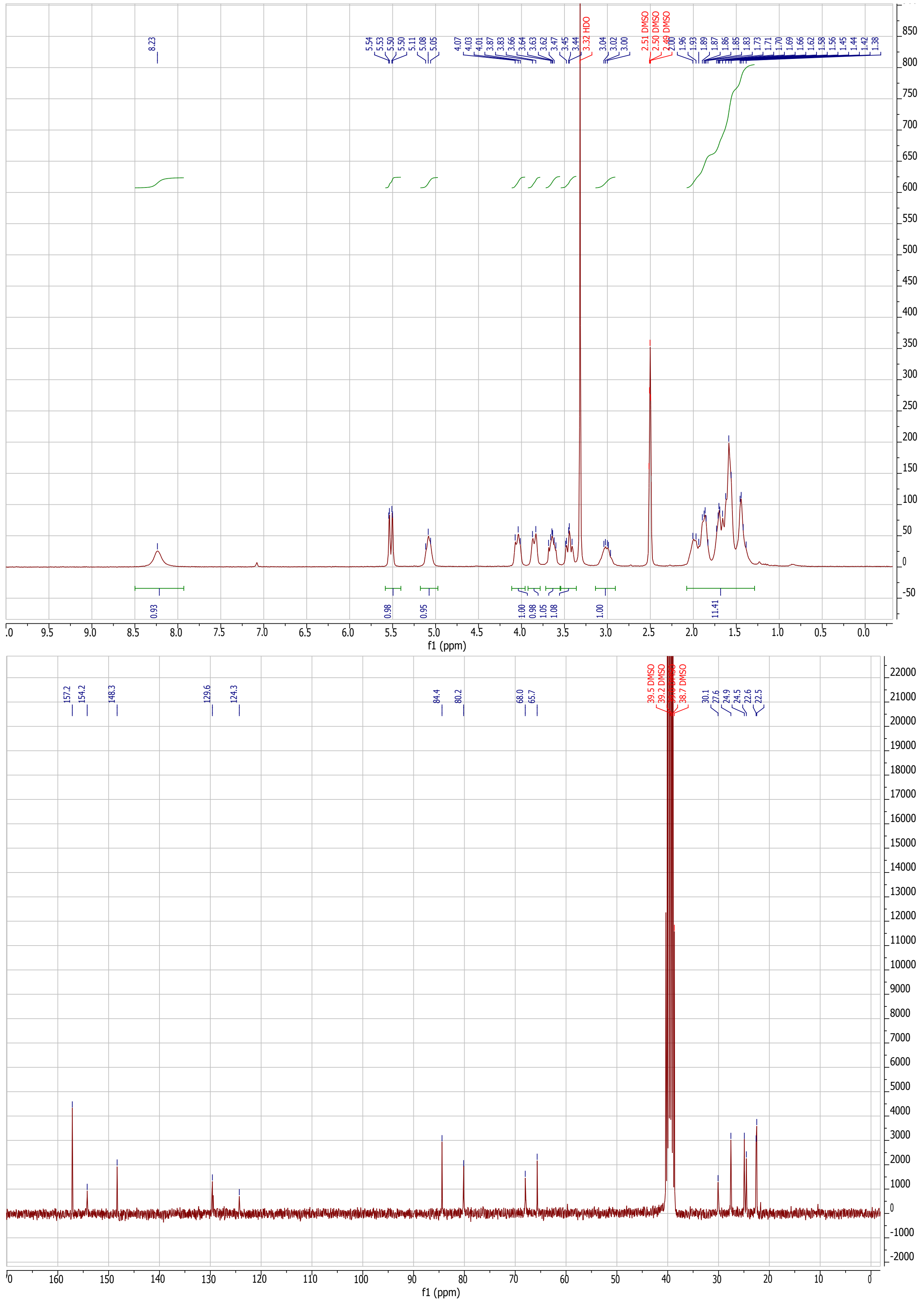

Figure S18. ^{1}H-NMR and ^{13}C-NMR 8-Bromo-6-chloro-*N*,9-bis(tetrahydro-2*H*-pyran-2-yl)-9*H*-purin-2-amine (**9**).

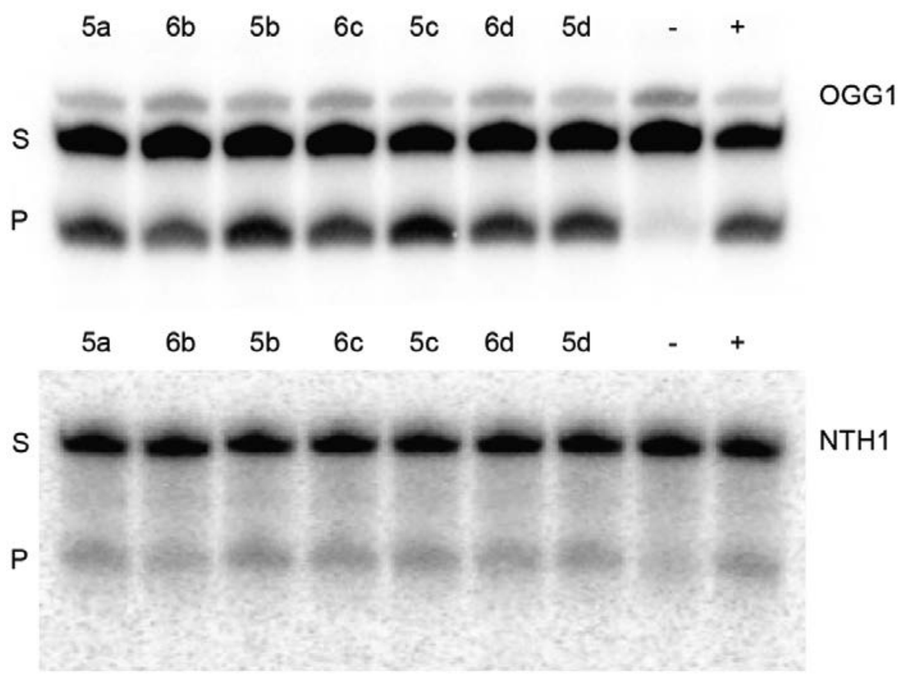

Figure S19. DNA glycosylase activity assays for OGG1 with 8oxoG substrate and NTH1 with 5-hydroxyuracil substrate in presence of compounds **5a**–**d** and **6b**–**d**. (**S**) uncleaved substrate; (**P**) cleaved product; (−) negative control; (+) positive control. Compounds were tested at 0.2 mM concentration for OGG1 and 0.5 mM for NTH1.
